# Donor Extended Blue TADF Dendrimer for High‐Performance Solution‐Processed OLEDs

**DOI:** 10.1002/advs.76130

**Published:** 2026-06-16

**Authors:** Mahni Fatahi, Yuka Yasuda, Ryo Kondo, Hironori Kaji, Eli Zysman‐Colman

**Affiliations:** ^1^ Organic Semiconductor Centre EaStCHEM School of Chemistry University of St Andrews St Andrews UK; ^2^ Institute for Chemical Research Kyoto University Kyoto Japan

**Keywords:** blue emission, dendrimer, reverse intersystem crossing, solution‐processed OLED, thermally activated delayed fluorescence

## Abstract

Achieving efficient and stable blue solution‐processed OLEDs remains an outstanding challenge in the field. We introduce a rational donor decoration strategy and apply it to the TADF emitter **DOBNA‐SpAc** (aka. **TDBA‐SAF**), exemplified in the emitter **DOBNA‐SpAc‐DCz**. By introducing *ter*(*tert*‐butylcarbazole) units at the 2 and 7 positions of the acridine moiety, solubility and hole‐transport properties are improved without compromising the blue emission endemic to **DOBNA‐SpAc**. This emitter has a high photoluminescence quantum yield, *Φ*_PL_, of 93% in 20 wt.% doped films in PPF (2,8‐bis(diphenyl‐phosphoryl)‐dibenzo[b,d]furan), a very small singlet‐triplet energy gap (Δ*E*_ST_ = 0.01 eV), and thus fast reverse intersystem crossing (*k*_RISC_ > 1 × 10^6^ s^−1^), resulting in a short delayed lifetime of 2 µs. Solution‐processed OLEDs with **DOBNA‐SpAc‐DCz** reached a maximum external quantum efficiency, *EQE*_max_, of 29.4 ± 0.1% at CIE coordinates of (0.145, 0.211). By probing different electron transport materials and comparing to devices using **DOBNA‐SpAc,** we found that the introduction of carbazole substituents promotes improved hole transport and a more spatially distributed recombination zone, while the faster *k*_RISC_ suppresses triplet‐related annihilation processes. These results demonstrate that the targeted peripheral donor dendron decoration of spiroacridine‐based TADF emitters is an effective strategy to achieve highly efficient emitters suitable for solution‐processed OLED applications.

## Graphical abstract

A dendronized MR‐TADF emitter, **DOBNA‐SpAc‐DCz**, was designed to improve solubility, hole transport, and exciton dynamics for solution‐processed OLEDs. Incorporation of *ter*(tert‐butylcarbazole) dendrons decreases Δ*E*_ST_ and accelerates RISC, yielding short delayed lifetimes (∼2 µs). The emitter affords efficient sky‐blue emission (474 nm) and enables high‐performance solution‐processed OLEDs with an *EQE*_max_ of 29.4%.

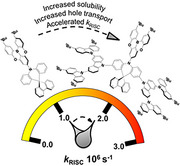

## 1 Introduction

Solution processing (SP) promises an attractive route toward cost‐efficient large‐area fabrication of organic light‐emitting diodes (OLEDs). High‐throughput techniques such as slot‐die coating and inkjet printing that are compatible with roll‐to‐roll manufacturing have been used to fabricate these devices [1, 2, 3]. Compared to vacuum deposition, SP uses less energy, and there is not as much material waste [2]. Yet, fabricating SP‐OLEDs that show comparable performance to vacuum‐deposited devices remains a challenge. What is required are high‐performance emitters optimized for solution‐processed devices, where solubility and film‐forming properties must be considered along with optoelectronic demands [2, 3].

Organic thermally activated delayed fluorescence (TADF) emitters are metal‐free and can harvest both singlet and triplet excitons, the latter via reverse intersystem crossing (RISC), enabling TADF OLEDs to reach comparably high internal quantum efficiencies as state‐of‐the‐art phosphorescent devices [4]. For high device performance, TADF emitters must have a fast RISC rate constant (*k*_RISC_) and a high photoluminescence quantum yield (*Φ*_PL_) [5]. Achieving these photophysical requirements while maintaining robust solution processability is non‐trivial, as molecular designs that minimize the singlet‐triplet energy gap (∆*E*_ST_) often compromise morphological stability or solubility as well as oscillator strength that adversely affects *Φ*_PL_ [6, 7]. Bridging this gap between excited‐state engineering and practical processability remains a central challenge for high‐efficiency SP‐OLEDs.

**FIGURE 1:**
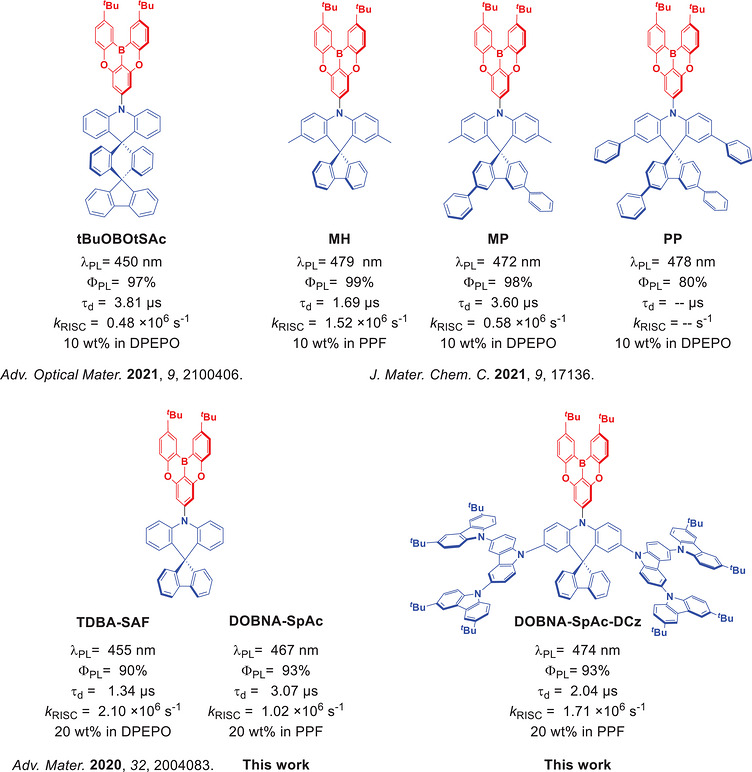
Chemical structures and key photophysical properties of DOBNA‐spiroacridine‐based TADF emitters and structure and key photophysical properties of DOBNA‐SpAc (aka TDBA‐SAF) and DOBNA‐SpAc‐DCz.

Among the different emission colors, optimizing the blue pixel is particularly challenging, particularly in terms of the required color point and the stability of the device [8]. Notably, several blue and deep‐blue TADF vacuum‐deposited OLEDs already demonstrate high efficiencies as a result of high *Φ*_PL_, small ∆*E*_ST,_ and fast *k*_RISC_ of the emitter. Of relevance to the current study, in 2020 Lim et al. reported **TDBA‐SAF** [9] (here referred to as **DOBNA‐SpAc**; Figure 1), which resembles a spiroacridine‐decorated derivative of the UV‐emitting compound **2a** [10], first reported by Hatakeyama et al. (later also referred to as **DOBNA** [11] or **BOO** [12]). By decorating the DOBNA core with the spiroacridine donor, the emission is red‐shifted to the deep blue, peaking at a *λ*_PL_ of 455 nm, and the *Φ*_PL_ of 90% remains high in 20 wt.% doped films in DPEPO (bis[2‐(diphenylphosphino)phenyl]ether oxide). A short delayed lifetime, *τ*_d_, of 1.34 µs means that *k*_RISC_ is fast at 2.10 × 10^6^ s^−1^. Vacuum‐deposited OLED reached a maximal external quantum efficiency (*EQE*_max_) of 28.2% at Commission Internationale de l’Éclairage (CIE) coordinates of (0.142, 0.090). Building on this promising work, Lee et al. designed a spiroacridine donor that contains a second spiro carbon‐linked fluorene group in an attempt to increase the horizontal alignment of the emitter and so enhance the light outcoupling in the device. Similar to **DOBNA‐SpAc**, **tBuOBOtSAc** [13] (Figure 1) emits in the deep blue at *λ*_PL_ of 450 nm with a higher *Φ*_PL_ of 97% in 10 wt.% doped films in DPEPO. However, this structural modification coincides with a longer *τ*_d_, to 3.81 µs, which contributes to the slower *k*_RISC_ of 0.48 × 10^6^ s^−1^ compared to **DOBNA‐SpAc**. Vacuum‐deposited OLEDs with **tBuOBOtSAc** showed a comparable *EQE*_max_ of 28.2% [CIE coordinates of (0.149, 0.061)] to those with **TDBA‐SAF**; however, they suffered from much stronger efficiency roll‐off.

Employing a different decoration strategy of the spiroacridine donor of **DOBNA‐SpAc**, Pei et al. introduced three derivatives, **MH**, **PH**, and **PP** (Figure 1) [14]. These three compounds contain methyl and/or phenyl groups at the 2, 3’, 6’, and 7 positions of the spiroacridine donor. The authors reported an apparent negative *∆E*_ST_ value for the 10 wt.% doped film **MH** in PPF, likely due to the measurements being conducted at 77 K, which is not low enough to completely suppress the delayed fluorescence in a compound that is expected to have a vanishingly small Δ*E*_ST_. The authors, however, found that when both the acridine and the fluorene moieties are decorated with phenyl groups as in **PP,** the *∆E*_ST_ is much larger at 200 meV. This trend coincides with that for *τ*_d_, where **MH** has the shortest delayed lifetime, followed by **MP**, whereas **PP** does not show any delayed emission in 10 wt.% doped films in PPF (2,8‐bis(diphenyl‐phosphoryl)‐dibenzo[b,d]furan), ostensibly due to non‐radiative decay outcompeting RISC. This study reveals that substitution at the fluorene moiety of the spiroacridine donor has an undesired impact on the photophysical properties of the emitter. The findings of these latter two studies reveal that only substitutions at the 2 and 7 positions of the spiroacridine of **DOBNA‐SpAc** produce a desirable change in the photophysical properties, such as an increase in *Φ*_PL_ and accelerated *k*_RISC_. On the contrary, substitution on the fluorene moiety leads to an increase in *τ*_d_ and subsequently a slower *k*_RISC_, rendering this substitution pattern unpromising in the light of device properties.

Inspired by these prior reports, we designed the blue‐emitting solution‐processable TADF emitter **DOBNA‐SpAc‐ DCz** (Figure 1) in which two *ter*(*tert*‐butylcarbazoles) decorate the spiroacridine donor at the 2 and 7 positions of the acridine. The motivation for the inclusion of these donor dendron units was to simultaneously decrease the *∆E*_ST_, improve the solubility and thus film‐forming properties, and enhance the hole transport as a counterbalance to the use of n‐type phosphine oxide‐based hosts. **DOBNA‐SpAc‐DCz** emits at 465 and 474 nm in toluene and 20 wt.% doped films in PPF, respectively, and has high *Φ*_PL_ of 79 and 93%, respectively. In both solution and the film state, the *τ*_d_ are short at around 2 µs, leading to fast *k*_RISC_ of 2.70 and 1.71 × 10^6^ s^−1^, respectively. These promising photophysical properties prompted the fabrication of solution‐processed OLEDs. The SP‐OLED emitted at 474 nm, corresponding to CIE coordinates of (0.145, 0.211), and showed an *EQE*_max_ of 29.4 ± 0.1%. In further device studies, using an altered device stack, a correlation between the increase in *k*_RISC_ and enhanced hole mobility of **DOBNA‐SpAc‐DCz** compared to **DOBNA‐SpAc** and reduced efficiency roll‐off was found.

## 2 Results and Discussion

### 2.1 Theoretical Calculations

**FIGURE 2:**
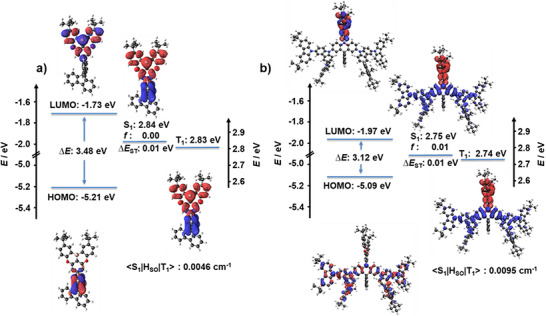
Calculated HOMO and LUMO energies and corresponding orbital plots (isovalue = 0.02), excited‐state energy levels and corresponding natural transition orbital plots and spin‐orbit coupling constants calculated on a PBE0/6‐31G(d,p) level of theory for (a) DOBNA‐SpAc and (b) DOBNA‐SpAc‐DCz.

To understand the interplay between the different fragments within the emitter design, we first performed calculations at a PBE0/6‐31G(d,p) level of theory in the gas phase. The HOMO/LUMO energy levels for **DOBNA‐SpAc‐DCz** are −5.09/−1.97 eV, and the orbital density plots show a clear separation of the HOMO, being located on the carbazole‐decorated spiroacridan moiety, and the LUMO, being located on the DOBNA unit (Figure 2). The HOMO of **DOBNA‐SpAc‐DCz** is destabilized compared to that of **DOBNA‐SpAc** (also known as **TDBA‐SAF**) at −5.21 eV, which is attributed to the extended π‐conjugated donor system. The LUMO is stabilized compared to that of **DOBNA‐SpAc** (−1.73 eV). A comparison of the calculated HOMO/LUMO levels to those of the structurally related compound **MH** (calculated at B3LYP/def2svp level of theory; Figure 1) reveals almost identical values for the LUMO energy (−1.99 eV) [14] and a stabilized HOMO for **DOBNA‐SpAc‐DCz** (**MH** HOMO: −4.98 eV) [14].

The excited‐state energies were calculated using TDA‐DFT at the same level of theory as the ground‐state optimized geometry. The energies of the S_1_ and the T_1_ states are predicted to be 2.75 and 2.74 eV, resulting in a ∆*E*_ST_ of 0.01 eV. The natural transition orbital visualizations indicate that the S_1_ state has long‐range charge transfer (LRCT) character between carbazole‐decorated spiroacridan, acting as the donor, and the DOBNA unit, acting as the acceptor. A very similar picture exists for the T_1_ state. Compared to **DOBNA‐SpAc** (S_1_/T_1_ = 2.84/2.83 eV), the S_1_ and T_1_ states are stabilized; however, a ∆*E*_ST_ of 0.01 eV remains the same for both emitters. The spin‐orbit coupling (SOC) between the T_1_ and S_1_ states is twofold stronger in **DOBNA‐SpAc‐DCz** (0.0095 cm^−1^) than in **DOBNA‐SpAc** (0.0046 cm^−1^), yet still weak. Notably, the root mean square difference for the geometry between the ground state (S_0_) and the optimised S_1_ state in both molecules remains low at 0.32 and 0.49 Å for **DOBNA‐SpAc** and **DOBNA‐SpAc‐DCz**, respectively, implying that the PL spectra of both should be equally narrowband (Figure S1). The ground‐state optimized conformation for both compounds reveals an expectedly large dihedral angle between the DOBNA and the spiroacridine unit, minimizing orbital overlap between the donor and acceptor moieties that explains the very small computed ∆*E*_ST_ (Figure S1).

### 2.2 Synthesis

**SCHEME 1:**
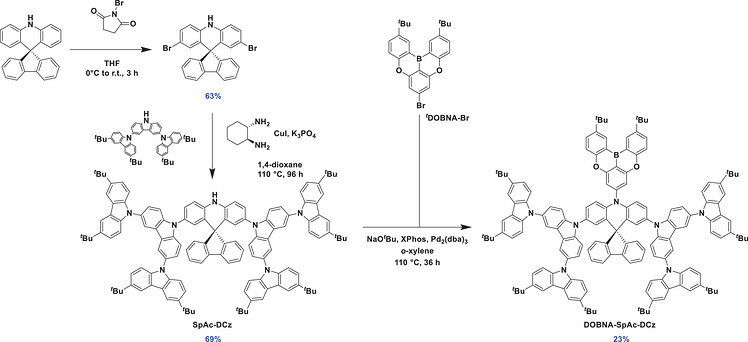
Synthetic route for DOBNA‐SpAc‐DCz.

The donor and acceptor moieties of **DOBNA‐SpAc‐DCz** were synthesised separately prior to being coupled together. The ***^t^* DOBNA‐Br** unit was obtained via a two‐step procedure following a previously reported protocol from the literature [9]. The donor moiety was synthesized in two steps from 10*H*‐Spiro[acridine‐9,9'‐fluorene], a dibromination at the 2‐ and 7‐positions of the acridine using *N*‐bromosuccinimide proceeded readily in 63% yield, followed by an Ullmann coupling with two equivalents of *ter*‐*tert‐*butylcarbazole to form the extended donor moiety **SpAc‐DCz** in 69% yield. **DOBNA‐SpAc‐DCz** was obtained in 23% yield following a Buchwald‐Hartwig type reaction between the donor and acceptor fragments (Scheme 1). The structure and purity were confirmed by ^1^H NMR and ^13^C NMR spectroscopy, high‐resolution mass spectrometry, melting point determination, and elemental analysis (Figures S14–S24). The high thermal stability was confirmed by thermal gravimetric analysis, which showed a 5% mass loss at a temperature *T*_d, 5%_ of 520°C (Figure S8), higher than that for **DOBNA‐SpAc** (*T*_d, 5%_: 400°C) [9], while the melting point for **DOBNA‐SpAc‐DCz** exceeded 400°C.

### 2.3 Photophysical Properties

**FIGURE 3:**
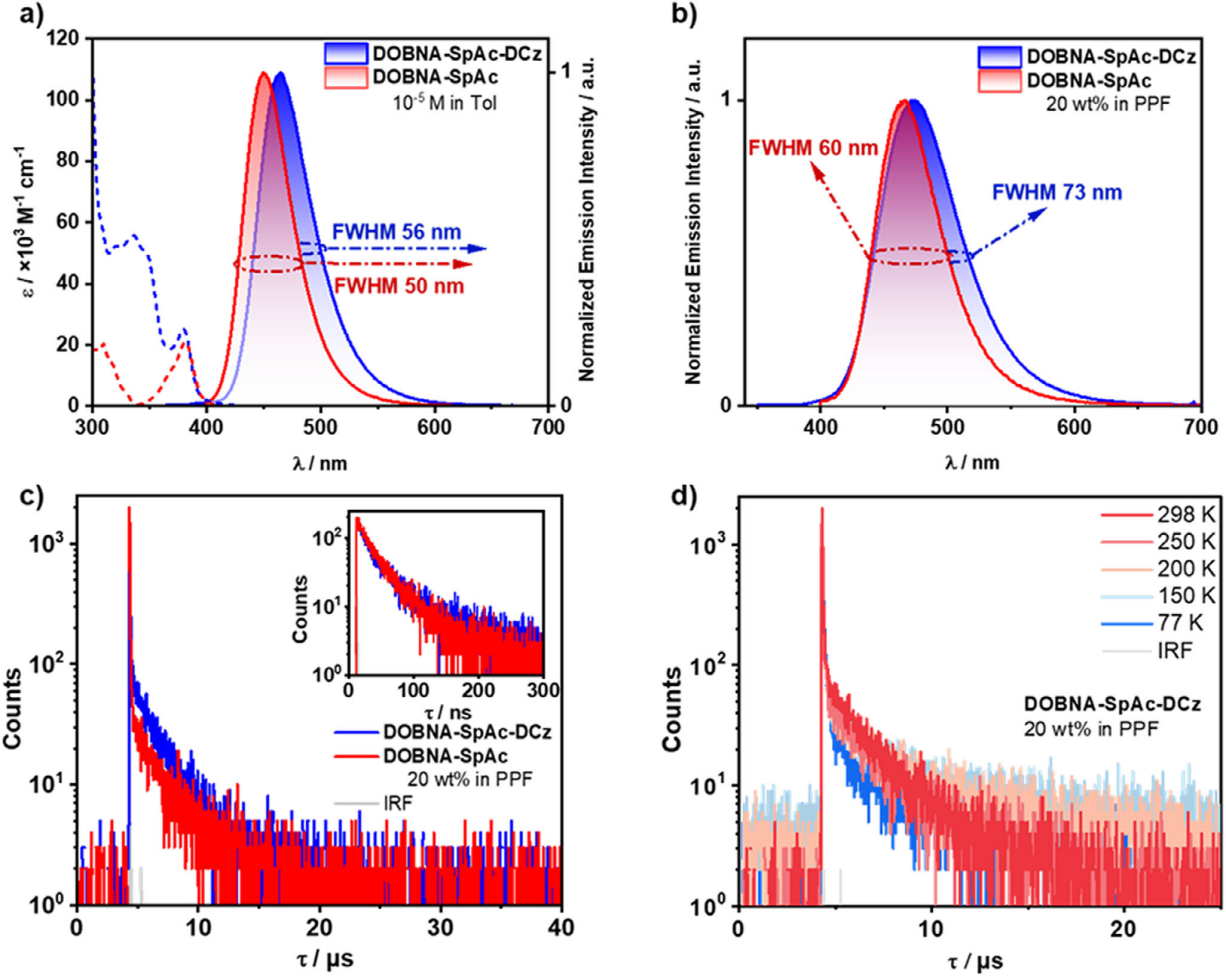
(a) Absorption (dashed) and normalized SS PL (solid) spectra of dilute toluene solutions of DOBNA‐SpAc‐DCz (blue; *λ*_exc_ = 350 nm) and DOBNA‐SpAc (red; *λ*_exc_ = 380 nm) (all 10^−5^ m); (b) Normalized SS PL spectra of 20 wt.% doped films of DOBNA‐SpAc‐DCz (blue) and DOBNA‐SpAc (red) in 2,8‐bis(diphenyl‐phosphoryl)‐dibenzo[b,d]furan (PPF); (*λ*_exc_ =  310 nm) (c) Time‐resolved PL (TRPL) decays of 20 wt.% doped films of DOBNA‐SpAc‐DCz (blue) and DOBNA‐SpAc (red) in PPF; Inset: TRPL decays showing the prompt PL; (d) Temperature‐dependent TRPL of a 20 wt.% doped film of DOBNA‐SpAc‐DCz in PPF.

The absorption and steady‐state photoluminescence (SS PL) spectra in dilute toluene solution of **DOBNA‐SpAc‐DCz** and **DOBNA‐SpAc** are shown in Figure 3a. The dominant low‐energy feature in the absorption spectra is a charge‐transfer band at 380 nm, with a molar extinction coefficient, ε, of 25 × 10^3^ m ^−1 ^cm^−1^ for the former, which effectively has about the same intensity as that in **DOBNA‐SpAc** (Table 1). The emission maximum of **DOBNA‐SpAc‐DCz**, *λ*_PL_, of 465 nm (FWHM of 56 nm) is slightly red‐shifted to that of **DOBNA‐SpAc** (*λ*_PL_ of 450 nm), in line with the computed trend. PL solvatochromism studies reveal that the PL red‐shifts and broadens, reflective of an emissive excited state of long‐range charge transfer (LRCT) character (Figure S4 and Table S2). The *Φ*_PL_ for **DOBNA‐SpAc‐DCz** is 79% in degassed toluene, which decreases significantly to 18% under aerated conditions, strongly indicating an involvement of triplet states in the emission mechanism. Time‐resolved PL measurements reveal a rather long prompt lifetime, *τ*_p_, of 14.6 ns and a prompt photoluminescence quantum yield, *Φ*_prompt_, of 12%, and a rather short delayed lifetime, *τ*_d_, of 2.47 µs and a delayed photoluminescence quantum yield, *Φ*_del_, of 67% (Figure S5). Excitonic rate constants were extracted from these measurements and are compiled in Table 1. The intersystem and reverse intersystem rate constants, *k*_ISC_ and *k*_RISC_, respectively, are fast at 5.59 × 10^7^ and 2.70 × 10^6^ s^−1^.

**TABLE 1:** Photophysical properties of dilute solutions of DOBNA‐SpAc‐DCz and 20 wt.% doped films of DOBNA‐SpAc‐DCz and DOBNA‐SpAc in PPF.

Compound	Medium	*λ*_Abs_ ^c^/nm	*λ*_PL_ ^d^ (FWHM)^e^/nm	*E*_S1_ ^f^ ^,g^/eV	*E*_T1_ ^f^ ^,g^/eV	*∆E*_ST_ ^h^/eV	*Φ*_PL_ ^i^/%	*τ*_p_ ^j^/ns	*τ*_d_ ^j^/µs	*k*_ISC_ ^k^/× 10^7^s^−1^	*k*_RISC_ ^k^/× 10^6^s^−1^	*k*_s_r_ ^k^/× 10^6^s^−1^	*k*_s_nr_ ^k^/× 10^6^s^−1^
**DOBNA‐SpAc‐DCz**	Sol.^a^	380 (25)	465(56)	2.93	2.92	0.01	79/18	14.6	2.47	5.59	2.70	8.12	2.16
	Film^b^	—	474(73)	2.90	2.90	<0.01	93/66	31.4	2.04	2.38	1.71	6.22	0.47
**DOBNA‐SpAc**	Sol.^a^	382 (21)	450(50)	3.02	2.93	0.09	—	—	—	—	—	—	—
	Film^b^	—	467(60)	2.91	2.91	<0.01	93/77	27.9	3.07	2.37	1.02	10.7	0.80

^a^ In toluene solutions (10^−5^ m).

^b^ Measured in spin‐coated thin films consisting of 20 wt.% emitter in PPF host.

^c^ Lowest energy absorbance peak, Absorptivity (*ε*) in parentheses (/10^3^ m ^−1^ cm^−1^).

^d^ Steady‐state emission maximum at 300 K. *λ*_exc_ = 350 nm (sol, **DOBNA‐SpAc‐DCz**), *λ*_exc_ = 380 nm (sol, **DOBNA‐SpAc**), *λ*_exc_ = 310 nm (film).

^e^ Full width at half maximum of emission peak.

^f^ S_1_ and T_1_ energies were obtained from the onsets of the respective steady state PL and phosphorescence spectra (delay: 1 ms (film)/10 ms (sol); gate time: 8.5 ms (film)/85 ms (sol)) at 77 K. *λ*_exc_ = 350 nm (sol, **DOBNA‐SpAc‐DCz**), *λ*_exc_ = 365 nm (sol, **DOBNA‐SpAc**), *λ*_exc_ = 310 nm (film).

^g^ Solution samples for Δ*E*_ST_ measurements were prepared in 2‐MeTHF (10^−6^ m).

^h^ Δ*E*_ST_ = *E*(S_1_)−*E*(T_1_).

^i^ Relative *Φ*_PL_ in solutions was measured by a comparative method using quinine sulfate as a standard (Φ_r_ = 54.6% in 1 N H_2_SO_4_) [17], while absolute *Φ*_PL_ of the thin films was measured using an integrating sphere.

^j^ Prompt and delayed lifetimes were obtained by TCSPC and MCS, respectively. *λ*_exc_ = 375 nm.

^k^ Intersystem and reverse intersystem crossing rate constants were calculated using the steady‐state approximation method as described in the literature [18].

The S_1_ and T_1_ energies were determined from the onsets of the SS PL and gated emission in 2‐methyltetrahydrofuran (2‐MeTHF) glass at 77 K (Figure S9) and are 2.93 and 2.92 eV, respectively; the corresponding ∆*E*_ST_ is 10 meV, matching the calculated value. By contrast, the S_1_ energy of **DOBNA‐SpAc** (Figure S9) is higher at 3.02 eV, while the T_1_ energy is almost unchanged at 2.93 eV, meaning that the ∆*E*_ST_ for this compound is larger at 90 meV.

Based on the promising photophysical properties in solution, we next investigated the photophysical properties of spin‐coated thin films. Here, we discuss the photophysical properties of 20 wt.% doped films of **DOBNA‐SpAc‐DCz** in a PPF matrix since this host‐guest combination has the highest *Φ*_PL_ (93% under nitrogen / 66% in air) of the tested OLED‐relevant hosts and doping concentrations (Figure S7 and Table S3). These films emit at *λ*_PL_ of 474 nm (FWHM of 73 nm). Compared to toluene, the PPF host provides a more polar local environment, which stabilizes the emissive excited state more strongly than the ground state. This stabilizes the S_1_ energy, which produces a red shift of the PL spectrum. The distribution of local microenvironments in the solid host (site‐to‐site variations in polarity and local electric fields) leads to inhomogeneous broadening, resulting in a wider emission band [15, 16]. In the film, the *τ*_p_ increased by a factor of two to 31.4 ns (*Φ*_prompt_ = 20%) compared to the solution‐state measurements (*τ*_p_ = 14.6 ns), while the *τ*_d_ remained unaffected (2.04 µs, *Φ*_del_ = 73%). The S_1_ and T_1_ energy levels, measured from the onsets of the steady‐state and time‐gated emission spectra at 77 K, are both 2.92 eV, resulting in an ∆*E*_ST_ that was too small to determine using our measurement setup. The negligible ∆*E*_ST_ is in good agreement with the solution‐state measurements as well as with the values reported for **tBuOBOtSAc** (0.08 eV, 10 wt.% in DPEPO) [13] and **MP** (0.02 eV, 10 wt.% in PPF) [14]. The excitonic rate constants for these films are similar to those for the solution‐state measurements; however, the non‐radiative decay rate constant from the singlet state, *k*_s_nr_, is different, where in the film it is smaller by a factor of 4, reflected in the higher *Φ*_PL_ (93 vs. 79%) compared to that in solution. The *k*_RISC_ is 1.71 × 10^6^ s^−1^. Compared to doped films of **DOBNA‐SpAc** at the same concentration, the *λ*_PL_ of **DOBNA‐SpAc‐DCz** is only slightly red‐shifted and broadened (Figure 3 and Table 1); the Δ*E*_ST_ is the same, while the *τ*_d_ decreased from 3.07 µs for **DOBNA‐SpAc** to 2.04 µs for **DOBNA‐SpAc‐DCz**. Although both emitters have the same *Φ*_PL_ of 93%, **DOBNA‐SpAc** shows a larger *Φ*_prompt_ (30%) and a smaller *Φ*_del_ (63%) compared to **DOBNA‐SpAc‐DCz** (20/73%), indicating more efficient triplet upconversion in the latter. The resulting *k*_RISC_ increases from 1.02 × 10^6^ s^−1^ for **DOBNA‐SpAc** to 1.71 × 10^6^ s^−1^ for **DOBNA‐SpAc‐DCz**. Temperature‐dependent TRPL measurements of both films reveal a weak temperature dependence of the delayed emission, confirming the TADF character of the emission (Figure 3d; Figure S6).

### 2.4 Electrochemistry

Cyclic voltammetry (CV) and differential pulse voltammetry (DPV) experiments in dichloromethane (DCM) using [*^n^* Bu_4_N]PF_6_ (0.1 m) as the supporting electrolyte and ferrocene/ferrocenium (Fc/Fc^+^) as an internal standard were used to infer the energies of the frontier molecular orbitals. The CVs of both **DOBNA‐SpAc‐DCz** and **DOBNA‐SpAc** show irreversible reduction waves at similar reduction potentials, *E*_red_, of −1.84 and −1.86 V vs. a standard calomel electrode (SCE) (Figure S3) [19]. The CVs of both compounds show quasi‐reversible oxidation waves, with the oxidation potential, *E*_ox_, of **DOBNA‐SpAc‐DCz** being cathodically shifted to 0.97 V vs. SCE compared to that of **DOBNA‐SpAc** (1.08 V vs. SCE) (Figure S3); this trend is in agreement with the DFT study of the HOMO levels of these two compounds. The LUMO energies for **DOBNA‐SpAc‐DCz** and **DOBNA‐SpAc** are similar at −2.50 and −2.49 eV, respectively, while the HOMO for **DOBNA‐SpAc‐DCz** (−5.31 eV) is destabilized compared to that of **DOBNA‐SpAc** (−5.42 eV). The corresponding HOMO and LUMO energies are in good agreement with the ones reported for **DOBNA‐SpAc** (aka. **TDBA‐SAF**) in the literature (−5.5/−2.4 eV) [9]. When compared to two other DOBNA‐containing emitters (**TBDA‐PAS** [20] and **BO‐tCzPhICz** [21]; Figure S2), the LUMO energies are similar (−2.44 [20] and −2.14 eV [21], respectively), indicating that the LUMO is located on the DOBNA moiety.

### 2.5 Device Properties

**FIGURE 4:**
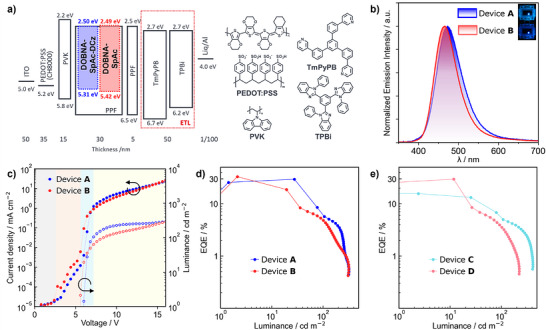
(a) Device structure and chemical structures of the materials used in solution‐processed OLEDs; (b) Normalized electroluminescence spectra of Device A (DOBNA‐SpAc‐DCz, blue) and Device B (DOBNA‐SpAc, red) employing TmPyPB as the electron‐transport layer; (c) Current density–voltage–luminance characteristics of Devices A and B; (d) External quantum efficiency‐luminance characteristics of Devices A and B; (e) External quantum efficiency‐luminance characteristics of Devices C and D employing TPBi as the electron‐transport layer.

Encouraged by the promising photophysical properties, we fabricated OLEDs using **DOBNA‐SpAc‐DCz** as the emitter. All devices were prepared by solution‐processing up to the emitting layer (EML). The detailed performance of the devices is summarized in Table 2. The device structures for Devices **A** and **B** were indium tin oxide (ITO, 50 nm)/poly(styrenesulfonic acid)‐doped poly(3,4‐ethylenedioxythiophene) (PEDOT:PSS (CH8000), 35 nm)/poly(N‐vinylcarbazole) (PVK, 15 nm)/EML (30 nm)/PPF (5 nm)/1,3,5‐Tris(3‐pyridyl‐3‐phenyl)benzene (TmPyPB, 35 nm)/8‐Quinolinolato lithium (Liq, 1 nm)/Al (100 nm) (Figure 4a). PEDOT:PSS CH8000 was employed as the hole‐injection layer due to its high work function, PVK as the hole‐transport and electron‐blocking layer, PPF as the electron‐transport and hole/exciton‐blocking layer, TmPyPB as the electron‐transport layer, and Liq as the electron‐injection layer. The EMLs consisted of PPF doped with 20 wt.% of the emitter; **DOBNA‐SpAc‐DCz** was used for Device **A**, while **DOBNA‐SpAc** was used for Device **B** as a reference.

**TABLE 2:** Device performances of solution‐processed OLEDs using DOBNA‐SpAc‐DCz and DOBNA‐SpAc as emitters.

Device	Emitter	Electron‐transporter	*λ*_EL_ ^a^ (FWHM)^b^/nm	*EQE* (Max/@100 cd m^−2^)^c^ (%)	*L*_max_ ^d^/cd m^−2^	*V*_on_ ^e^/V	CIE (x, y)^f^
**A**	**DOBNA‐SpAc‐DCz**	TmPyPB	474(73)	29.4 ± 0.1/5.3 ± 0.5	287 ± 36	6.0	(0.145, 0.211)
**B**	**DOBNA‐SpAc**	TmPyPB	466(68)	33.9 ± 0.9/4.9 ± 0.1	321 ± 0	5.6	(0.140, 0.136)
**C**	**DOBNA‐SpAc‐DCz**	TPBi	473(71)	13.2 ± 2.8/5.0± 1.0	400 ± 18	6.8	(0.147, 0.231)
**D**	**DOBNA‐SpAc**	TPBi	474(66)	28.7 ± 0.9/3.5 ± 0.1	229 ± 4	6.4	(0.141, 0.143)

^a^ Electroluminescence peak wavelength.

^b^ Full width at half maximum.

^c^ Average external quantum efficiency of two devices at the maximum value and at 100 cd m**^−^** ^2^.

^d^ Average maximum luminance of two devices.

^e^ Turn‐on voltage at 1 cd m^−2^.

^f^ CIE coordinates at 1 mA cm^−2^.

Devices **A** and **B** exhibited blue emission with peak wavelengths, *λ*_EL_, of 474 and 464 nm, respectively, which are in good agreement with the *λ*_PL_ values of the corresponding doped films (Figures 4b and 3b). The current density‐voltage plots of Devices **A** and **B** clearly show four distinct regimes (Figure 4c). Such behavior was not observed in devices employing other combinations of hole‐transport (i.e., OTPB and X‐DCDPA in Device **Z**; Figure S10) and host materials (i.e., CzSi in Device **X** or DPEPO in Device **Y**; Figure S10), indicating that this phenomenon is specific to the interface between PVK and the phosphine‐oxide‐based host material, PPF. Because the LUMO levels of the ETL and the EML are well aligned, electron injection into the EML is efficient. Consequently, at low driving voltages, electrons tend to accumulate near the PVK/PPF interface. By contrast, the large mismatch between the HOMO levels of PVK and PPF is likely to promote localization of hole traps at their interface. As the applied voltage increases, these interfacial hole traps are progressively filled, and once they are filled, holes begin to flow rapidly into the EML. As a result, the accumulated electrons and the injected holes undergo rapid recombination. Exceptionally high *EQE*_max_ values of 29.4 ± 0.1% for Device A and 33.9 ± 0.9% for Device B were obtained (Figure 4d). However, localized charge accumulation and concentrated recombination also induce a pronounced efficiency roll‐off at high luminance and current density in both devices. Since both devices reached nearly the same maximum luminance, the observed efficiency roll‐off was attributed not to emitter‐specific processes but rather to the host material common to both devices.

To isolate the impact of emitter modification on device performance, additional Devices **C** and **D** were fabricated by replacing TmPyPB with TPBi (2,2′,2''‐(1,3,5‐Benzinetriyl)‐tris(1‐phenyl‐1‐*H*‐benzimidazole)) as the electron‐transport layer (Figure S12). TPBi has an electron mobility about two orders of magnitude lower than that of TmPyPB [22, 23]. These devices were designed to reduce the electron density at low operating voltages, thereby suppressing electron accumulation at the interface. The maximum luminance of Device **C** increased to 400 ± 18 cd m^−2^, and the efficiency roll‐off was suppressed compared to the corresponding Device **D** (Figure 4e). Moreover, the introduction of additional carbazole units in **DOBNA‐SpAc‐DCz** enhances the hole‐transport properties, leading to a more spatially distributed recombination zone within the EML (Figure S11). In addition, the fast RISC of **DOBNA‐SpAc‐DCz** reduces the triplet exciton density, thereby suppressing exciton‐exciton and exciton‐charge annihilation processes that are responsible for efficiency roll‐off [5].

## 3 Conclusions

In this work, we demonstrated that by introducing donor dendrons onto the spiroacridine moiety of the blue‐emitting donor‐acceptor TADF emitter, **DOBNA‐SpAc**, an acceleration of *k*_RISC_ in **DOBNA‐SpAc‐DCz** was achieved. **DOBNA‐SpAc‐DCz** possesses a very small Δ*E*_ST_ of less than 10 meV and short delayed lifetimes of 2 µs, resulting in high *k*_RISC_ values of > 1 × 10^6^ s^−1^ in both toluene solution and 20 wt.% doped film in PPF, making it a promising candidate for solution‐processed OLEDs. The solution‐processed OLEDs with **DOBNA‐SpAc‐DCz** and **DOBNA‐SpAc** showed high *EQE*_max_ of 29.4 ± 0.1% and 33.9 ± 0.9% at CIE coordinates of (0.145, 0.211) and (0.140, 0.136), respectively. The pronounced efficiency roll‐off observed in Devices **A** and **B** originates predominantly from interfacial charge accumulation at the PVK/PPF interface rather than intrinsic emitter limitations, as evidenced by the modified device architecture employing TPBi. Notably, the additional carbazole units and faster RISC of **DOBNA‐SpAc‐DCz** promote improved hole transport and reduced triplet exciton accumulation, enabling a more balanced recombination profile. This leads to a reduced efficiency roll‐off, and the device using **DOBNA‐SpAc‐DCz** exhibits a higher *EQE*_100_ of 5.0 ± 0.1% and a higher maximum luminance of 400 ± 18 cd m^−2^ compared to **DOBNA‐SpAc** (*EQE*_100_ = 3.5 ± 0.1%; *L*_max_ = 229 ± 4 cd m^−2^).

## Author Contributions

M.F.: Conceptualization, investigation, methodology, validation, formal analysis, visualization, writing – original draft, writing – review & editing. Y.Y.: Investigation, writing – original draft, writing – review & editing. R.K.: Investigation, writing – review & editing. H.K.: Project administration, resources, supervision, writing – review & editing. E.Z‐C.: Validation, formal analysis, project administration, resources, supervision, writing – review & editing.

## Conflicts of Interest

The authors declare no conflicts of interest.

## Supporting information




**Supporting File 1**: advs76130‐sup‐0001‐SuppMat.pdf.

**Supporting File 2**: advs76130‐sup‐0002‐Data.zip.

## Data Availability

The research data supporting this publication can be accessed at https://doi.org/10.17630/c9c7dd21‐ddb8‐4868‐a3fa‐1e9224ced324.
