# Supplementary material for: Donor Extended Blue TADF Dendrimer for High‐Performance Solution‐Processed OLEDs
Source: Adv Sci (Weinh). 2026 Jun 16:e76130. Online ahead of print. doi: 10.1002/advs.76130 (PMC13336851; doi:10.1002/advs.76130)
Supplement: Supplementary file 1 — Supporting File 1: advs76130‐sup‐0001‐SuppMat.pdf. [file ADVS-9999-e76130-s001.pdf]

Donor extended blue TADF dendrimer for high-performance solution-processed OLEDs

Mahni Fatahi‡[a], Yuka Yasuda‡[b], Ryo Kondo[b], Hironori Kaji[b] and Eli Zysman-Colman*[a]*

[a]Organic Semiconductor Centre, EaStCHEM School of Chemistry, University of St Andrews, St Andrews, UK, KY16 9ST. E-mail: eli.zysman-colman@st-andrews.ac.uk

[b]Institute for Chemical Research, Kyoto University, Gokasho, Uji, Kyoto 611-0011, Japan. E-mail: kaji@scl.kyoto-u.ac.jp

‡ These authors contributed equally

Table of Contents

General Synthetic Procedures S2

Quantum chemical calculations S5

Photophysical measurements S5

Electrochemistry measurements S7

Fitting of time-resolved photoluminescence measurements S8

Kinetics rate constant calculations S8

OLED Fabrication and Characterization S9

Experimental Section

General Synthetic Procedures. The following starting materials were synthesized according to literature materials, **tDOBNA-Br**,[1] ***ter*-tCz**,[2] and **TBDA-SAF** (**DOBNA-SpAc**).[1] All other reagents and solvents were obtained from commercial sources and used as received. Air-sensitive reactions were performed under a nitrogen atmosphere using Schlenk techniques, no special precautions were taken to exclude air or moisture during work-up and crystallisation. Anhydrous THF was obtained from an MBraun SPS5 solvent purification system. Flash column chromatography was carried out using silica gel (Silia-P from Silicycle, 60 Å, 40-63 μm). Flash column chromatography was carried out using a Teledyne ISCO CombiFlash® NextGen 300+ system with RediSep® Normal-Phase Silica columns in sizes of 24 - 80 g silica packing. Analytical thin-layer-chromatography (TLC) was performed with silica plates with aluminum backings (250 μm with F-254 indicator). TLC visualization was accomplished using a 254/365 nm UV lamp. HPLC analysis was conducted on a Shimadzu LC-40 HPLC system. GPC-HPLC was performed using a Shim-pack GPC-803 column. ^{1}H and ^{13}C NMR spectra were recorded on a Bruker Advance spectrometer (500 MHz for ^{1}H, 125 MHz for ^{13}C). The following abbreviations have been used for multiplicity assignments: "s" for singlet, "d" for doublet, "dd" for doublet of doublets, "td" for triplet of doublets and "m" for multiplet, ^{1}H and ^{13}C NMR spectra were referenced residual solvent peaks with respect to TMS (δ = 0 ppm). Melting points were measured using open-ended capillaries on an Electrothermal 1101D Mel-Temp apparatus and are uncorrected. High-resolution mass spectrometry (HRMS) was performed on a MALDI-TOF/TOF (Bruker Daltonics) at University of Edinburgh Mass Spectrometry Facility. Elemental analysis was performed by Ms. Orla McCullough , London Metropolitan University.

2,7-dibromo-10H-spiro[acridine-9,9'-fluorene] (SpAc-Br)

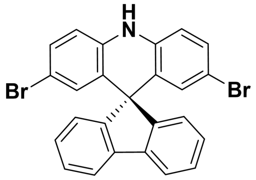

In a flask wrapped in aluminum foil was charged with 10H-spiro[acridine-9,9'-fluorene] (0.33 g, 1.00 mmol, 1.0 equiv.) in THF (40 mL). A separate foil-wrapped flask charged with N-bromosuccinimide (0.39 g, 2.20 mmol, 2.2 equiv.) in THF (20 mL) was transferred to the first flask portion-wise with a pipette over a period of 20 min. After the addition was complete, the reaction was stirred at room temperature for 3 h. After complete consumption of 10H-spiro[acridine-9,9'-fluorene] the reaction was quenched with water (50 mL) and extracted with DCM (3 × 60 mL). The combined organic layers were washed with sat. aq. sodium thiosulfate solution and sat. brine. The organic layers were then dried over $MgSO_4$, filtered and the solvent was removed under reduced pressure. The crude product was purified by washing with cold hexane (50 mL). The product was obtained as a grey solid. **Yield**: 63% (0.31 g). **R*f***: 0.34 (EtOAc/Hex30/70% on silica gel). **Mp**: 293-297 °C. **^{1}H NMR (500 MHz, DMSO) δ (ppm)**: 9.67 (s, 1H), 7.97 (d, *J* = 7.6 Hz, 2H), 7.43 (td, *J* = 7.5, 1.2 Hz, 2H), 7.29 (td, *J* = 7.5, 1.1 Hz, 2H), 7.26 – 7.21 (m, 4H), 6.96 (d, *J* = 8.6 Hz, 2H), 6.12 (d, *J* = 2.3 Hz, 2H). **^{13}C NMR (126 MHz, DMSO) δ (ppm)**: 155.32, 138.76, 138.67, 131.03, 129.35, 129.16, 128.85, 125.70, 125.42, 121.15, 117.20, 111.10, 56.31. **HR-MS** (**MALDI-MS**) **[M+H]$^+$ Calculated:** ($C_{25}H_{15}Br_2N$) 488.95453; **Found:** 488.95075.

2,7-bis(3,3'',6,6''-tetra-*tert*-butyl-9'H-[9,3':6',9''-tercarbazol]-9'-yl)-10H-spiro[acridine-9,9'-fluorene] (SpAc-DCz)

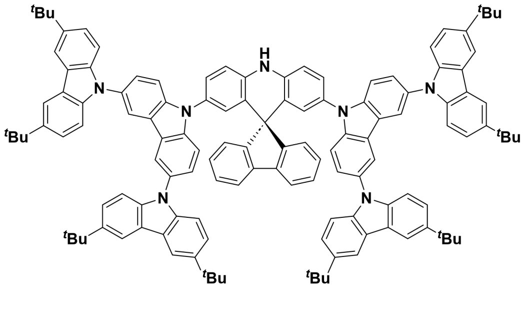

In a Schlenk flask charged with nitrogen **SpAc-Br** (0.20 g, 0.41 mmol, 1.0 equiv.), 3,3'',6,6''-tetra-*tert*-butyl-9'H-9,3':6',9''-tercarbazole (0.65 g, 0.90 mmol, 2.2 equiv.), K_3PO_4 (0.87 g, 4.09 mmol, 10 equiv.) copper(I) iodide (4 mol%) evacuated for 3 × 30 seconds. Afterwards (1S,2S)-cyclohexane-1,2-diamine (7 mol%) was added and all reagents were dissolved in anhydrous 1,4-dioxane (12 mL). The reaction mixture was heated to 110 °C

and stirred for 72 h. After complete consumption of **(1)** the reaction was quenched with water and extracted with DCM (3x 80 mL). The combined organic layers were washed with sat. brine, dried over $MgSO_4$ and filtered before the solvent was removed under reduced pressure. The crude product was purified by flash chromatography (Hex/DCM 85/15%) and by GPC (Biobeads SX-3 resin; eluent inhibitor-free THF). The product was obtained as an off-white solid. **Yield**: 69% (0.50 g). **R*f***: 0.51 (EtOAc/Hex30/70% on silica gel). **Mp**: >400 °C. **^{1}H NMR (500 MHz, CD_2Cl_2) δ (ppm)**: 8.16 (dd, *J* = 16.1, 1.9 Hz, 12H), 7.76 – 7.70 (m, 2H), 7.66 – 7.58 (m, 2H), 7.51 (dd, *J* = 8.5, 2.4 Hz, 2H), 7.45 (ddd, *J* = 8.7, 6.8, 2.0 Hz, 12H), 7.38 – 7.34 (m, 4H), 7.32 (d, *J* = 8.7 Hz, 4H), 7.28 (d, *J* = 8.6 Hz, 8H), 7.20 (d, *J* = 8.5 Hz, 2H), 6.91 (s, 1H), 6.76 (d, *J* = 2.3 Hz, 2H), 1.45 (s, 72H). **^{13}C NMR (126 MHz, CD_2Cl_2) δ (ppm)**: 156.86, 143.18, 140.78, 140.64, 139.79, 138.50, 130.99, 130.51, 129.16, 128.80, 127.35, 126.71, 126.14, 125.65, 124.16, 123.54, 121.01, 119.58, 116.75, 116.34, 111.54, 109.66, 57.41, 35.18, 32.34. **HR-MS (MALDI-MS) Calculated**: ($C_{129}H_{122}N_7$) 1770.9867 **Found**: 1770.9935.

10-(2,12-di-*tert*-butyl-5,9-dioxa-13b-boranaphtho[3,2,1-de]anthracen-7-yl)-2,7-bis(3,3'',6,6''-tetra-*tert*-butyl-9'H-[9,3':6',9''-tercarbazol]-9'-yl)-10H-spiro[acridine-9,9'-fluorene] (DOBNA-SpAc-DCz)

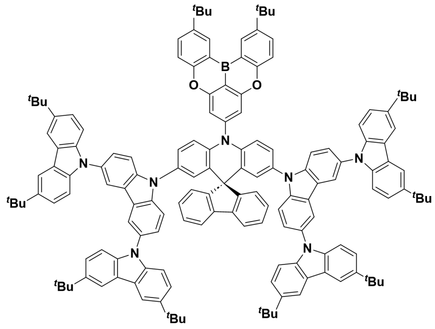

A Schlenk flask charged with nitrogen **SpAc-DCz** (0.18 g, 0.10 mmol, 1.0 equiv.), 7-bromo-2,12-di-*tert*-butyl-5,9-dioxa-13b-boranaphtho[3,2,1-de]anthracene (***^{t}*DOBNA-Br**) (0.05 g, 0.10 mmol, 1.0 equiv.), NaO*^{t}*Bu (0.03 g, 0.30 mmol, 3.0 equiv.), Xphos (0.01 g, 0.02 mmol, 0.15 equiv.), and Pd_2dba_3 (0.01 g, 0.01 mmol, 0.1 equiv.) was evacuated for 3 × 30 seconds. Afterwards, anhydrous *o*-xylene (7 mL) was added, and the mixture was heated to 110 °C and stirred for 36 h. The reaction was quenched with water (20 mL) and extracted with DCM (3x 50 mL). The combined organic layers were washed with sat. brine, dried over $MgSO_4$, filtered and the solvent removed under reduced pressure. The crude product was purified by flash chromatography (Hex/DCM 92.5/7.5%) and GPC (Biobeads SX-3 resin; eluent inhibitor-free THF). The

product was obtained as a light-yellow solid. **Yield**: 23% (0.05 g). **R*f***: 0.35 (EtoAc/Hex 15/85% on silica gel). **Mp**: >400 °C. **^{1}H NMR (500 MHz, CD_2Cl_2) δ (ppm)**: 8.93 (d, *J* = 2.4 Hz, 2H), 8.16 (dd, *J* = 7.5, 1.7 Hz, 12H), 7.93 (dd, *J* = 8.8, 2.4 Hz, 2H), 7.85 (dd, *J* = 12.0, 7.5 Hz, 4H), 7.71 – 7.62 (m, 4H), 7.54 – 7.40 (m, 18H), 7.35 (d, *J* = 8.7 Hz, 4H), 7.28 (d, *J* = 8.6 Hz, 8H), 6.99 (d, *J* = 8.9 Hz, 2H), 6.89 (d, *J* = 2.4 Hz, 2H), 1.56 (s, 18H), 1.45 (s, 72H). **^{13}C NMR (126 MHz, CD_2Cl_2) δ (ppm)**: 160.53, 159.38, 157.38, 146.74, 146.44, 143.13, 140.62, 140.58, 139.88, 132.83, 131.08, 130.99, 130.78, 129.34, 128.90, 127.17, 126.49, 126.28, 126.13, 124.18, 124.13, 123.50, 122.19, 121.17, 119.54, 118.56, 117.33, 116.73, 115.81, 111.55, 111.36, 109.63, 57.37, 35.15, 32.31, 31.86. **HR-MS (MALDI-MS) Calculated**: ($C_{155}H_{148}BN_7O_2$) 2151.1827 **Found**: 2151.1808. **Elemental analysis**: **Calculated for $C_{155}H_{148}BN_7O_2$**: C: 86.52 H: 6.93 N: 4.56 **Found**: C: 86.30 H: 6.14 N: 4.25. **HPLC-GPC:** 100.00% pure, retention time 9.177 min in 100% THF.

Quantum chemical calculations. Density functional theory (DFT) and TD(A)-DFT calculations were performed using the Gaussian 16 revision C.01 package.[3] The ground-state optimization was carried out using DFT using the PBE0[4] functional, the 6-31G(d,p) basis set,[5] and in conjunction with the D3(BJ)[6] dispersion correction scheme in the gas phase, starting from a structure drawn and optimized using Chem3D.[7] The vertical excited-state calculations were performed using Time-Dependent DFT within the Tamm-Dancoff approximation (TDA-DFT),[8] with the same functional and basis set as for the ground-state geometry optimization in the gas phase. Spin-orbit coupling matrix elements SOCME were calculated based on the optimized ground state geometry using PySOC.[9] The molecular orbital distributions were visualized with Gaussview 6.0.[7] The RMSD of the S_0 and S_1 states were visualized using the VMD program.[10] Calculations were submitted and processed using the Digichem software package (version 7),[11-12] which incorporates a number of publicly available software libraries, including: cclib[13] for parsing of result files, VMD[10]/Tachyon[14] for 3D rendering, Matplotlib for drawing of graphs[15], Open Babel[16]/Pybe[17] file interconversion.

Photophysical measurements. Optically dilute solutions of concentrations on the order of 10^{-5} or 10^{-6} M were prepared in spectroscopic-grade toluene for absorption and photoluminescence (PL) spectral analysis. Absorption spectra were recorded at room

temperature on a Shimadzu UV-2600 double beam spectrophotometer with a 1 cm quartz cuvette. Molar absorptivity determination was verified by linear regression analysis of values obtained from at least four independent solutions at varying concentrations ranging from 3.0×10^{-6} to 1.0×10^{-5} M with absorbance ranging from 0.025 to 0.100.

For emission studies, aerated solutions, steady-state PL and excitation spectra and time-resolved PL measurements were recorded at room temperature using an Edinburgh Instruments FS5 fluorimeter. Samples were excited at 350 nm for steady-state PL measurements and 375 nm for time-resolved PL decays. Degassed solutions were prepared via three freeze-pump-thaw cycles and spectra were measured using a home-made Schlenk quartz cuvette. Photoluminescence quantum yields (Φ_{PL}) for solutions were determined using the optically dilute method[18] in which four sample solutions with absorbances of ca. 0.10, 0.095, 0.080 and 0.06 at 350 nm were used. The Beer-Lambert law was found to remain linear across the concentrations of the solutions. For each sample, linearity between absorption and emission intensity was verified through linear regression analysis with the Pearson regression factor (R^2) for the linear fit of the data set surpassing 0.9. Individual relative quantum yield values were calculated for each solution and the values reported represent the slope obtained from the linear fit of these results. The Φ_{PL} was determined using the equation $\Phi_{PL} = (\Phi_r * \frac{A_r}{A_s} * \frac{I_s}{I_r} * \frac{n_s^2}{n_r^2})$, where A stands for the absorbance at the excitation wavelength (λ_{exc}: 350 nm), I is the integrated area under the corrected emission curve and n is the refractive index of the solvent with the subscripts “s” and “r” representing sample and reference respectively. Φ_r is the absolute PL quantum yield of the external reference, quinine sulfate (Φ_r = 54.6% in 1 *N* H_2SO_4).[19] The experimental uncertainty in the Φ_{PL} is conservatively estimated to be 10%, though we have found that statistically we can reproduce Φ_{PL} values to within 3% relative error.

For solid-state film measurements, the 20 wt% doped films of emitters in a host matrix were prepared from solution which were prepared as follows, 80% w/w (8 mg) of host was dissolved in 0.4 mL of solvent and to this, 20% w/w (2 mg emitter in 100 μL of solvent) of emitter was added. Thin films were then spin-coated onto a sapphire substrate using a spin speed of 1500 rpm for 60 s and annealed at 50 °C for 60 s to obtain a thickness of ~80

nm. An integrating sphere (Edinburgh Instruments FS5, SC30 module) was employed for the photoluminescence quantum yield measurements of thin film samples. The Φ_{PL} of the films were then measured in air and in N_2 by purging the integrating sphere with N_2 gas flow for 2 min. The photophysical properties of the film samples were measured using an Edinburgh Instruments FS5 fluorimeter. Time-resolved PL measurements of the thin films were carried out using the multi-channel scaling (MCS) and time-correlated single-photon counting (TCSPC) technique. The samples were excited at 375 nm by a pulsed laser and were kept in a vacuum of $< 1 \times 10^{-4}$ mbar.

The singlet and triplet state energies in 2-MeTHF glass and in doped film were determined from the onset values of the steady-state PL (SS PL) and delayed emission spectra at 77 K. The singlet-triplet energy gap (ΔE_{ST}) was estimated from the difference in energy of the onsets of these spectra. For SS PL, samples were excited by a xenon lamp emitting at 365 nm (2-MeTHF) and 310 nm (20 wt% in PPF). For phosphorescence spectra, samples were excited by a xenon flashlamp emitting at 365 nm (2-MeTHF) and 310 nm (20 wt% in PPF) (EI FS5, SC-70). Phosphorescence spectra were measured with a time-gated window of 10-85 ms (2-MeTHF) and 1-8.5 ms (20 wt% in PPF) with xenon flashlamp operating at 10 Hz (2-MeTHF) and 100 Hz (20 wt% in PPF).

Electrochemistry measurements. Cyclic Voltammetry (CV) analysis was performed on an Electrochemical Analyzer potentiostat model 620E from CH Instruments at a sweep rate of 100 mV/s. Differential pulse voltammetry (DPV) was conducted with an increment potential of 0.004 V and a pulse amplitude, width, and period of 50 mV, 0.05, and 0.5 s, respectively. Samples were prepared as dichloromethane (DCM) solutions, which were degassed by sparging with DCM-saturated nitrogen gas for 5 minutes prior to measurements. All measurements were performed using 0.1 M DCM solution of tetra-*n*-butylammonium hexafluorophosphate ($[^nBu_4N]PF_6$). An Ag/Ag^+ electrode was used as the reference electrode, while a platinum electrode and a platinum wire were used as the working electrode and counter electrode, respectively. The redox potentials are reported relative to a saturated calomel electrode (SCE) with a ferrocenium/ferrocene (Fc/Fc^+) redox couple as the internal standard (0.46 V vs SCE).[20]

Fitting of time-resolved photoluminescence measurements: Time-resolved PL measurements were fitted to a sum of exponentials decay model, with chi-squared (χ^2) values between 1 and 2, using the Fluoracle software. Each component of the decay is assigned a weight, (w_i), which is the contribution of the emission from each component to the total emission.

The average lifetime was then calculated using the following:

- Two exponential decay model:

$$\tau_{AVG} = \tau_1 w_1 + \tau_2 w_2$$

with weights defined as $w_1 = \frac{A1\tau_1}{A1\tau_1 + A2\tau_2}$ and $w_2 = \frac{A2\tau_2}{A1\tau_1 + A2\tau_2}$ where A1 and A2 are the preexponential-factors of each component.

- Three exponential decay model:

$$\tau_{AVG} = \tau_1 w_1 + \tau_2 w_2 + \tau_3 w_3$$

with weights defined as $w_1 = \frac{A1\tau_1}{A1\tau_1 + A2\tau_2 + A3\tau_3}$, $w_2 = \frac{A2\tau_2}{A1\tau_1 + A2\tau_2 + A3\tau_3}$ and $w_3 = \frac{A3\tau_3}{A1\tau_1 + A2\tau_2 + A3\tau_3}$ where A1, A2 and A3 are the preexponential-factors of each component.

Kinetics rate constant calculations

The kinetics constants in this paper are calculated following the literature:[21]

$k_p = 1/\tau_p$, $k_d = 1/\tau_d$

$$k_r^S = k_p \Phi_{PF}$$

$$k_{nr}^S = k_p \frac{\Phi_{PF}}{\Phi_{PLQY}} (1 - \Phi_{PLQY})$$

$$k_{nr}^{T} = k_d(1 - \frac{\Phi_{DF}}{1 - \Phi_{PF}})$$

$$k_{ISC} = \{[k_p(1 - \Phi_{PF}) - k_d\Phi_{PF}]\Phi_{PLQY} + k_p\Phi_{PF}\Phi_{DF} + [k_p\Phi_{PF}^{2}(1 - \Phi_{PLQY}) + k_d\Phi_{DF}\Phi_{PLQY}]\}/(2\Phi_{PF}\Phi_{PLQY})$$

$$k_{RISC} = \frac{\Phi_{PLQY}}{2} \times \frac{\Phi_{PLQY}(1 - \Phi_{PF}) + \Phi_{DF} + \Phi_{PF}(1 - \Phi_{PLQY})}{\Phi_{PF}(1 - \Phi_{PF})}$$

$$K_{eq} = \frac{3k_{RISC} + k_r^T + k_{nr}^T}{3(k_r^s + k_{nr}^s) + k_{ISC}}$$

For a TADF emitter with photoluminescence quantum yield near unity and no phosphorescence contribution,

$$k_r^S K_{eq} = \frac{4k_r^S k_{RISC}}{3k_r^S + 4k_{ISC}}$$

OLED Fabrication and Characterization

The solution-processed devices were fabricated as follows. Patterned ITO substrates were irradiated with UV-O_3 for 30 min. PEDOT:PSS (CH 8000) was diluted by mixed with ultra-pure water (1:1) and the mixture was spin-coated onto the ITO substrate at 500 rpm for 1 s. 500 rpm for 1 s, 4000 rpm for 12 s, and then 500 rpm for 1 s followed by annealing at 150 °C for 10 min in air. Then, PVK dissolved in *o*-dichlorobenzene (10 mg mL^{-1}) was spin-coated at 2000 rpm for 30 s and baked at 120 °C for 10 min in air. The emitters and host materials dissolved in chlorobenzene (10 mg/mL), were spin-coated and annealed at 100 °C for 10 min under vacuum. After that, the remaining layers, PPF (5 nm), TmPyPB or TPBi (50 nm), Liq (1 nm) were deposited in in inorganic chamber under 10^{-5} Pa. Finally, Al (100 nm) was deposited in inorganic chamber under 10^{-4} Pa. Vacuum evaporation was carried out using SE-4260 (ALS Technology, Japan). Device characterization was carried out using an absolute EQE measurement system (C9920-12, Hamamatsu Photonics, Japan)

equipped with a source meter (2400, Keithley, Japan). These measurements were conducted in the forward direction in 400 mV steps.

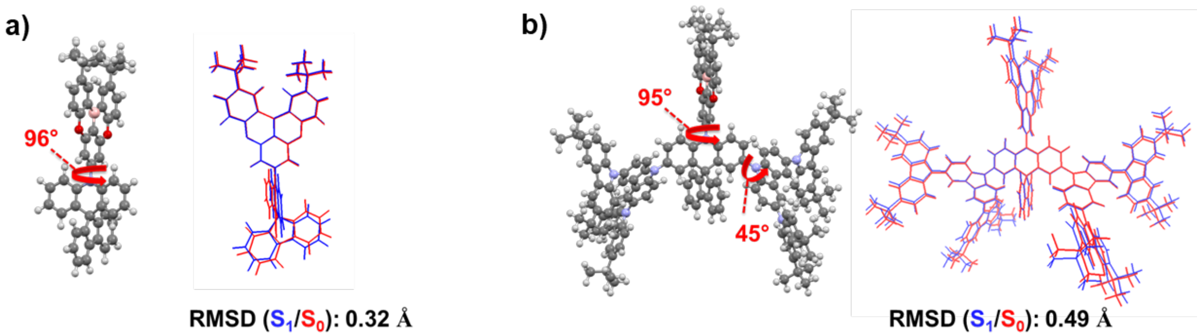

Figure **S1**. Visualization of dihedral angles between donor and acceptor and RMSD visualization for S_1 and ground state geometry and corresponding RMSD value in Å for a) **DOBNA-SpAc** and b) **DOBNA-SpAc-DCz**.

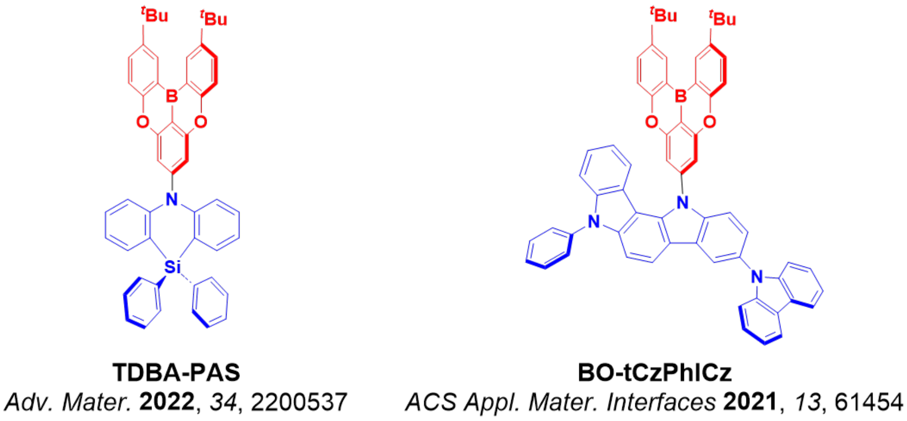

Figure **S2**. Chemical structures of **TDBA-PAS**[22] and **BO-tCzPhICz**.[23]

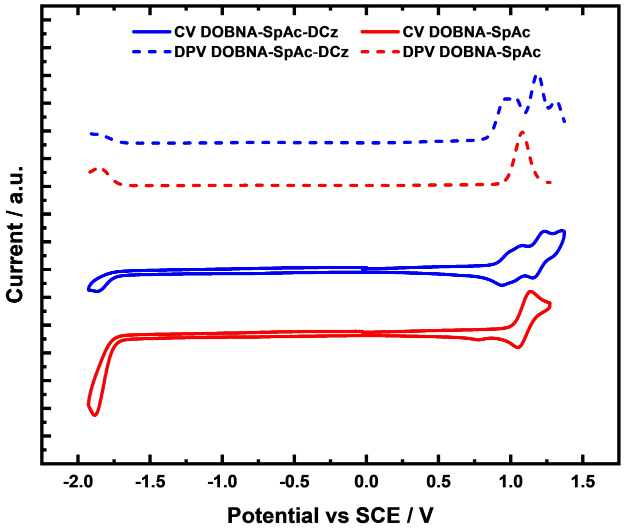

Figure **S3**. CV (solid) and DPV (dashed) of **DOBNA-SpAc-DCz** (blue) and **DOBNA-SpAc** (red). CV and DPV performed in DCM solution (0.1 M [^{n}Bu$_4$N]PF$_6$) with a scan rate of 0.1 V/s and referenced to SCE ($E_{ox/red}$ [V *vs.* SCE] = $E_{ox/red}$ [V *vs.* Fc/Fc$^+$] + 0.46).

Table **S1**. Oxidation and reduction potentials and calculated HOMO and LUMO energy levels for **DOBNA-SpAc-DCz** and **DOBNA-SpAc**.

	$E_{ox}{}^a$ / V	$E_{red}{}^a$ / V	$E_{HOMO}{}^b$ / eV	$E_{LUMO}{}^c$ / eV	$\Delta E_{HOMO\text{-}LUMO}{}^d$ / eV
**DOBNA-SpAc-DCz**	0.97	-1.84	-5.31	-2.50	2.81
**DOBNA-SpAc**	1.08	-1.86	-5.42	-2.49	2.94

^{a} Oxidation and reduction potential derived from peaks from the DPV performed in DCM solution (0.1 M [^{n}Bu$_4$N]PF$_6$) with a scan rate of 0.1 V/s and referenced to [Fc/Fc$^+$]$^+$. ^{b} HOMO energy calculated from -(E_{ox}+4.8). ^{c} LUMO energy calculated from -(E_{red}+4.8). ^{d} $\Delta E_{HOMO/LUMO}$ = | E_{HOMO} - E_{LUMO}|.

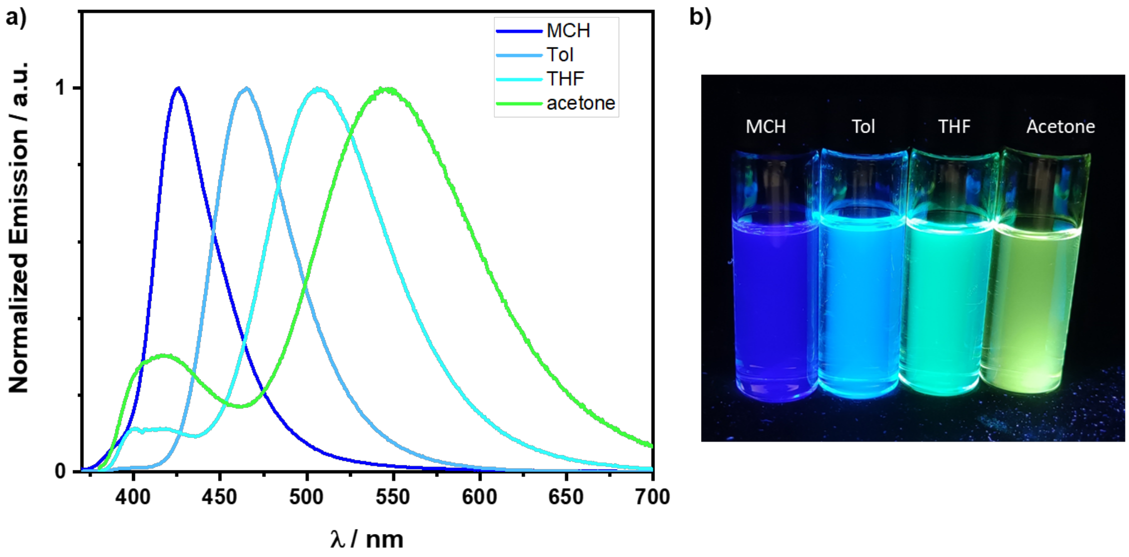

Figure **S4**. Solvatochromism PL study using solvents with increasing polarity from methylcyclohexane (MCH) to acetone. a) Steady-state PL spectra (λ_{exc}= 365 nm); b) Photo of measured solutions under UV-irradiation. (Conc.: 10^{-5} M)

Table **S2**. Photophysical properties of dilute solution of **DOBNA-SpAc-DCz** in solvents with increasing polarity from methyl-cyclohexane to acetone.

Solvent^{a}	λ_{PL}^{b} / nm	FWHM^{b} / nm
Methyl-cyclohexane	426	43
Toluene	465	56
THF	(400), 506	83
Acetone	(417), 543	107

^{a} at a concentration of 10^{-5} M; ^{b} λ_{exc}= 365 nm.

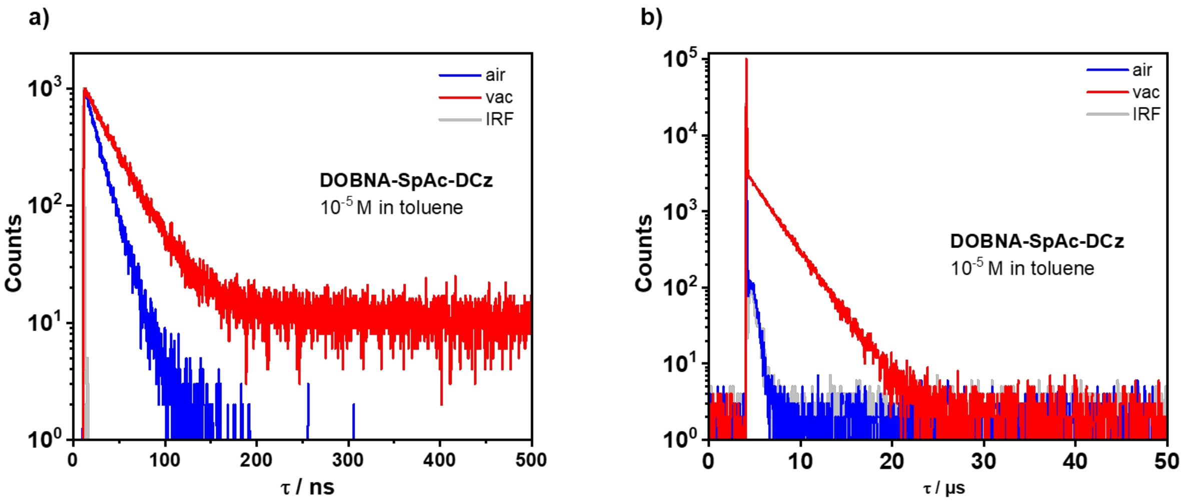

Figure **S5**. TRPL decays of dilute toluene solution of **DOBNA-SpAc-DCz** (10^{-5} M); a) prompt fluorescence decay measured by TCSPC (λ_{exc}= 375 nm); b) delayed fluorescence decay measured by MCS (λ_{exc}= 375 nm).

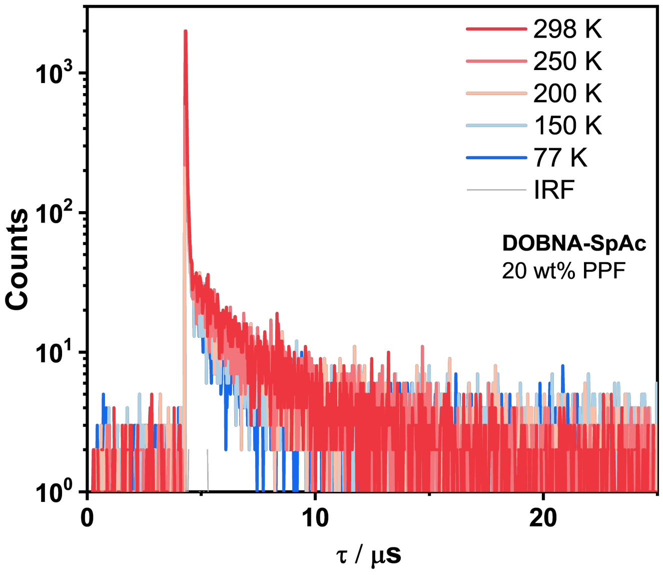

Figure **S6**. Temperature-dependent TRPL of a 20 wt% doped film of **DOBNA-SpAc** in PPF. (λ_{exc}= 375 nm).

Table **S3**. Photoluminescence quantum yield of **DOBNA-SpAc-DCz** in various OLED hosts.

Host	λ_{exc} / nm	Doping %	Φ_{PL} N_2 /%	$\Phi_{PL\ air}$ /%
**2,6DCzPPy**	310	5	24	20
		10	28	23
		20	30	25
**DOBNA-Tol**	310	5	37	33
		10	37	33
		20	34	30
**HT3:ET7 1:1**	310	5	29	22
		10	37	23
		20	35	25
**mCP**	310	10	35	28
		20	37	26
**mCP:PPT 1:1**	310	5	43	33
		10	52	40
		20	51	39
**PPT**	310	5	73	61
		10	77	61
		20	51	46
**DPEPO**	310	10	65	36
**PPF**	310	5	72	52
		10	76	57
		20	93	66
		30	86	59

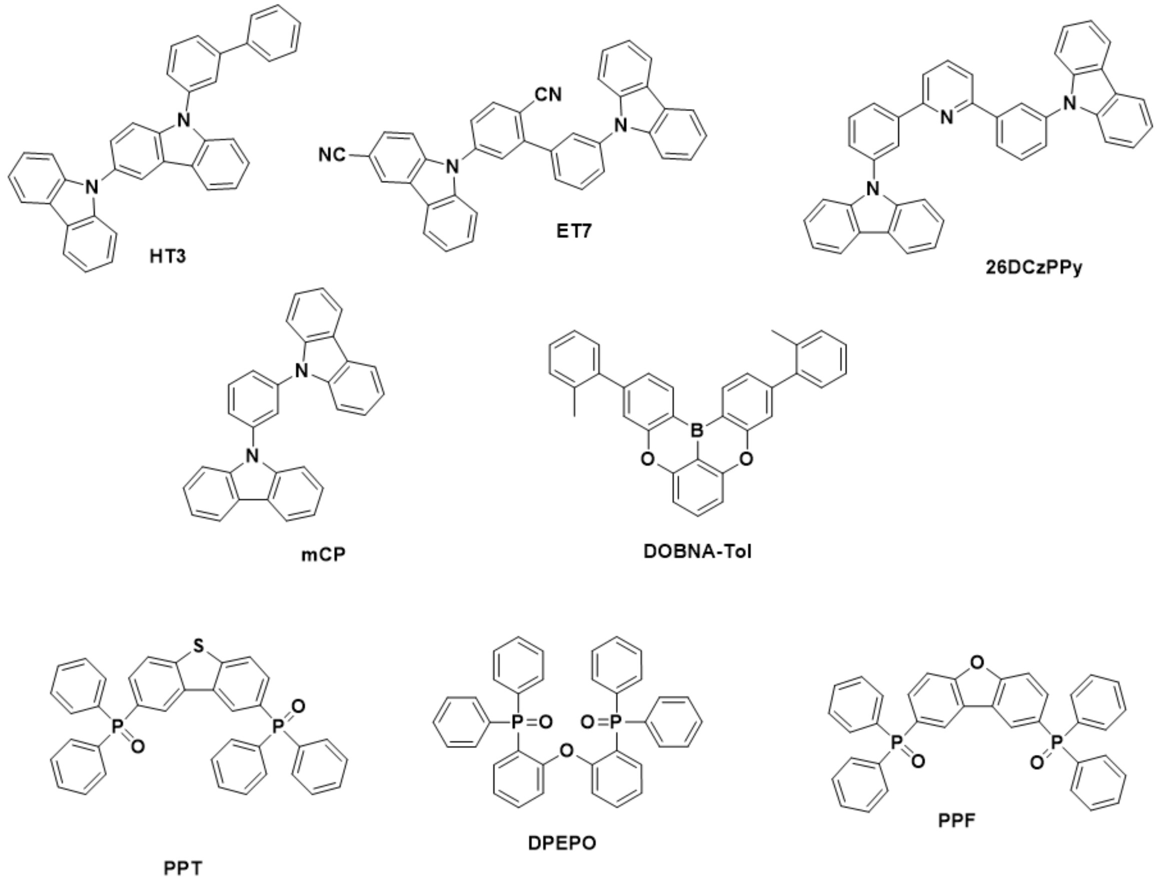

Figure **S7**. Chemical structures of tested host materials in Table **S3**.

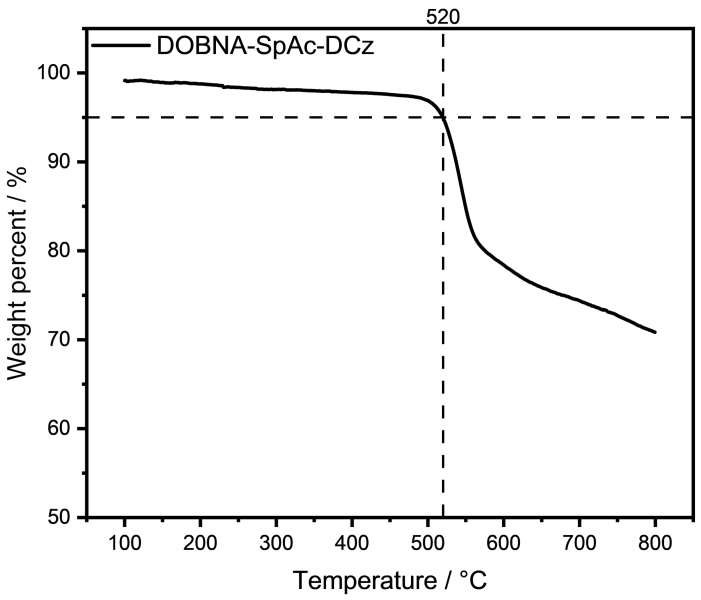

Figure **S8**. Thermogravimetric analysis of **DOBNA-SpAc-DCz**.

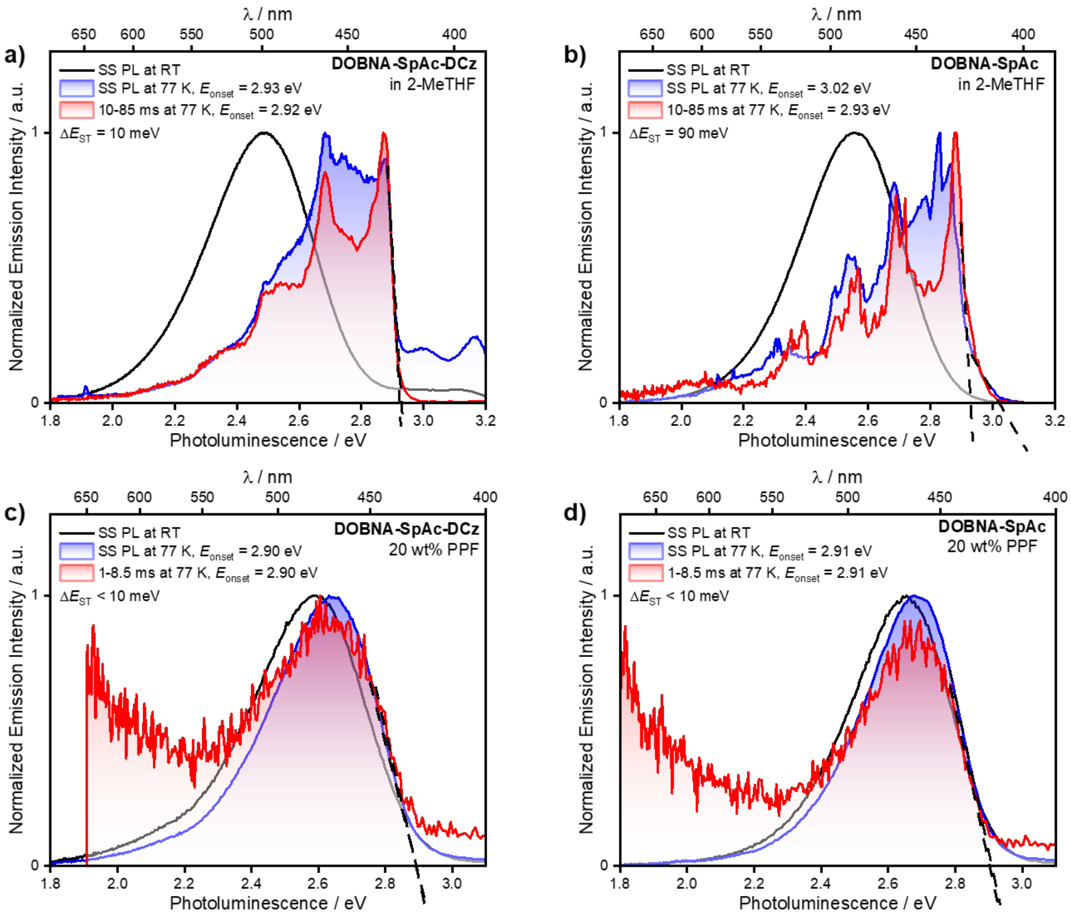

Figure **S9**. Steady state PL at room temperature (black), steady state PL at 77 K (blue), gated emission at 77 K (red) of a) **DOBNA-SpAc-DCz** in 2-MeTHF (10^{-5} M, λ_{exc}= 365 nm); b) **DOBNA-SpAc** in 2-MeTHF (10^{-5} M, λ_{exc}= 380 nm); c) 20 wt% **DOBNA-SpAc-DCz** in PPF (λ_{exc}= 310 nm); d) 20 wt% **DOBNA-SpAc** in PPF (λ_{exc}= 310 nm).

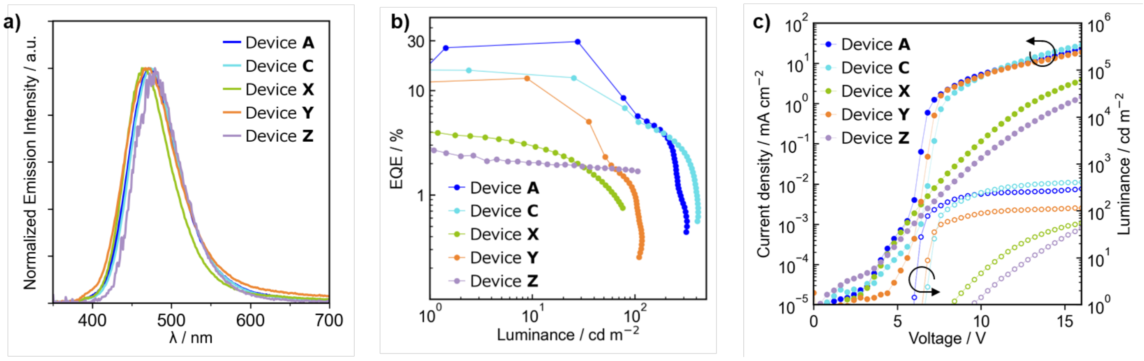

Figure **S10**. a) Normalized electroluminescence; b) External quantum efficiency-luminance plot; c) Current density–voltage–luminance plot.

The structures of the respective devices with **DOBNA-SpAc-DCz** are as follows.

Device **A**: ITO/PEDOT:PSS/PVK/Emitter in PPF/PPF/TmPyPB/Liq/Al;

Device **C**: ITO/PEDOT:PSS/PVK/Emitter in PPF/PPF/**TPBi**/Liq/Al;

Device **X**: ITO/PEDOT:PSS/PVK/Emitter in **CzSi**/PPF/BmPyPhB/Liq/Al;

Device **Y**: ITO/PEDOT:PSS/PVK/Emitter in **DPEPO**/PPF/ BmPyPhB /Liq/Al;

Device **Z**: ITO/PEDOT:PSS/**OTPD/X-DCDPA**/Emitter in PPF/PPF/TmPyPB/Liq/Al;

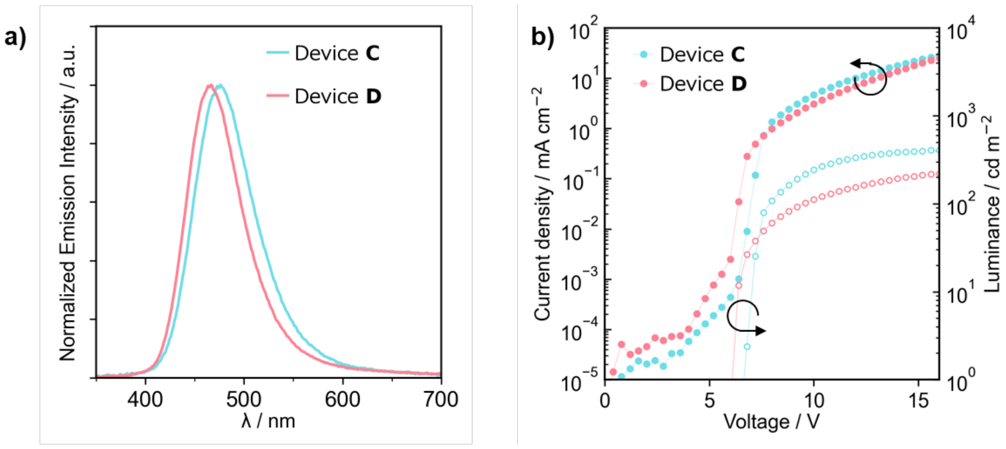

Figure **S11**. a) Normalized electroluminescence; b) Current density–voltage–luminance plot plot of Device **C** and **D**.

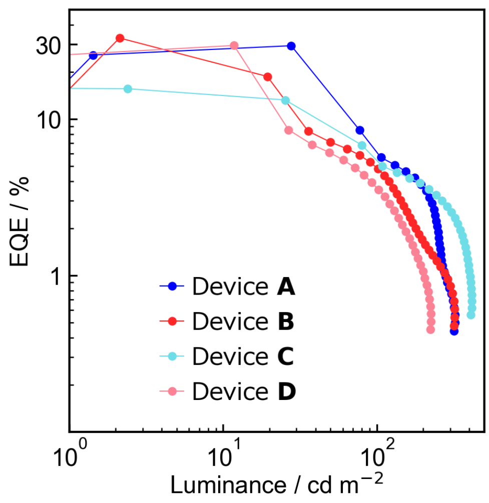

Figure **S12**. Comparison of the external quantum efficiency-luminance plot for Devices **A**, **B**, **C**, and **D**.

Table **S4**. Device data for repeat devices of structure **A**, **B**, **C**, and **D.**

Device	Emitter	Electron -transporter	λ_{EL} [a] (FWHM)[b] / nm	*EQE* (Max/@100 cd m^{-2}) [c] (%)	L_{max} [d] / cd m^{-2}	V_{on} [e] / V	CIE (x, y) [f]
**A**	**DOBNA-SpAc-DCz**	TmPyPB	474 (73)	29.4/5.7<br>29.3/4.8	322<br>251	6.0<br>6.0	(0.145, 0.211)
**B**	**DOBNA-SpAc**	TmPyPB	466 (68)	33.0/4.8<br>34.8/4.9	321<br>321	5.6<br>5.6	(0.140, 0.136)
**C**	**DOBNA-SpAc-DCz**	TPBi	473 (71)	15.8/5.0<br>10.6/4.9<br>15.5/2.2	417<br>382<br>144	6.8<br>6.8<br>6.4	(0.147, 0.231)
**D**	**DOBNA-SpAc**	TPBi	474 (66)	29.5/3.5<br>27.8/3.4	225<br>233	6.4<br>6.4	(0.141, 0.143)

[a] Electroluminescence peak wavelength. [b] Full width at half maximum. [c] external quantum efficiency at the maximum value and at 100 cd m^{-2}. [d] maximum luminance. [e] Turn-on voltage at 1 cd m^{-2}. [f] CIE coordinates at 1 mA cm^{-2}.

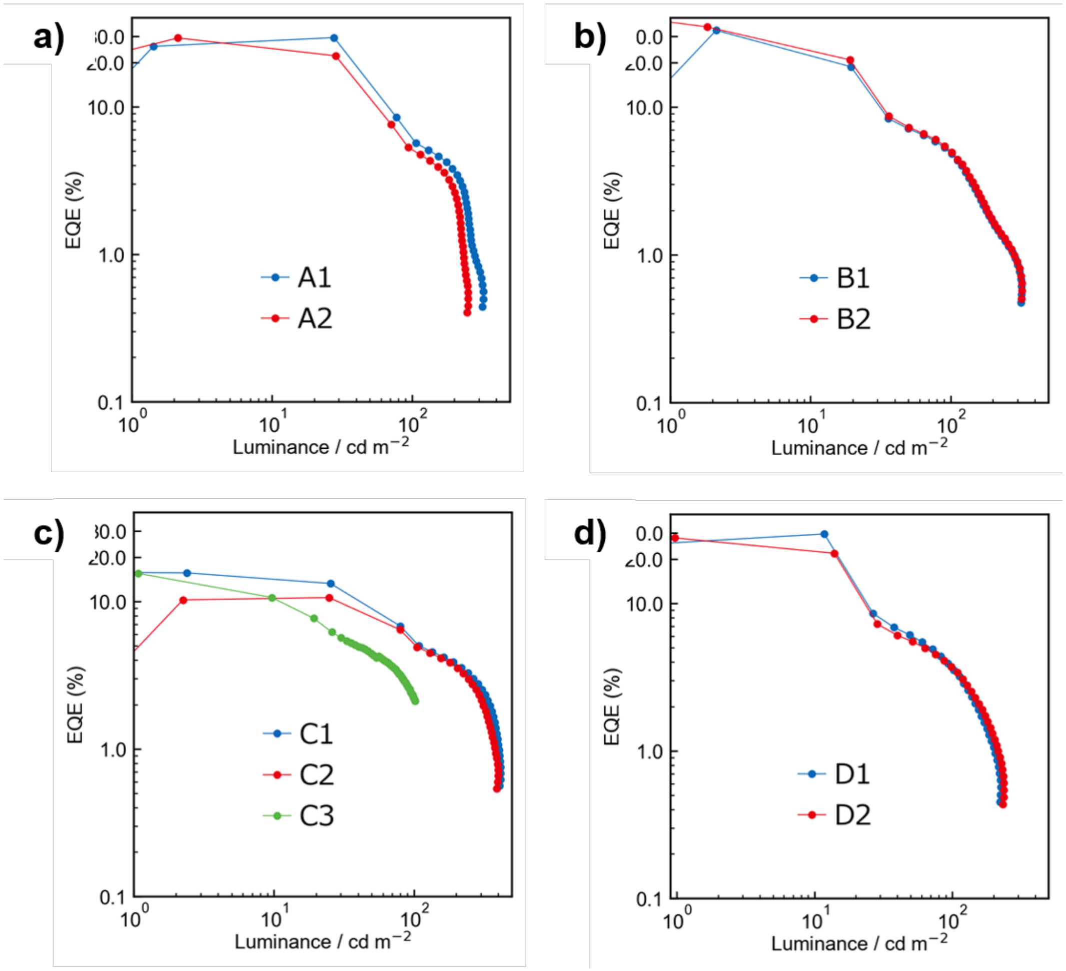

Figure **S13**. External quantum efficiency-luminance plot for repeat devices of a) Device **A**, b) Device **B**, c) Device **C**, and d) Device **D**.

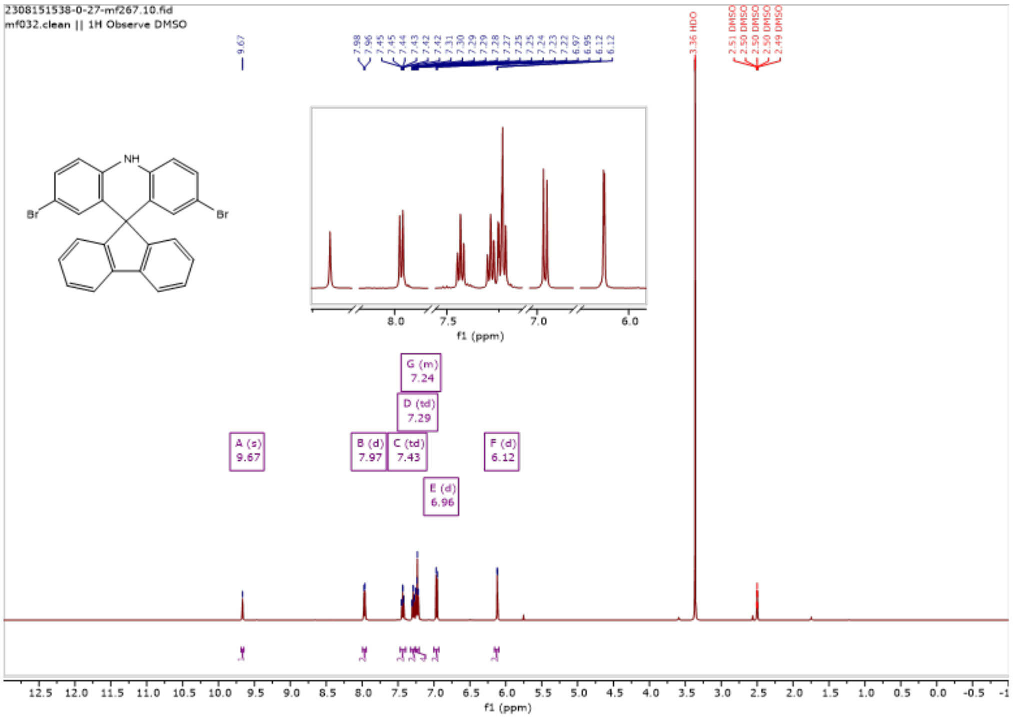

Figure **S14**. ^{1}H-NMR spectrum of **2,7-dibromo-10*H*-spiro[acridine-9,9'-fluorene] (SpAc-Br)** in DMSO-d_6.

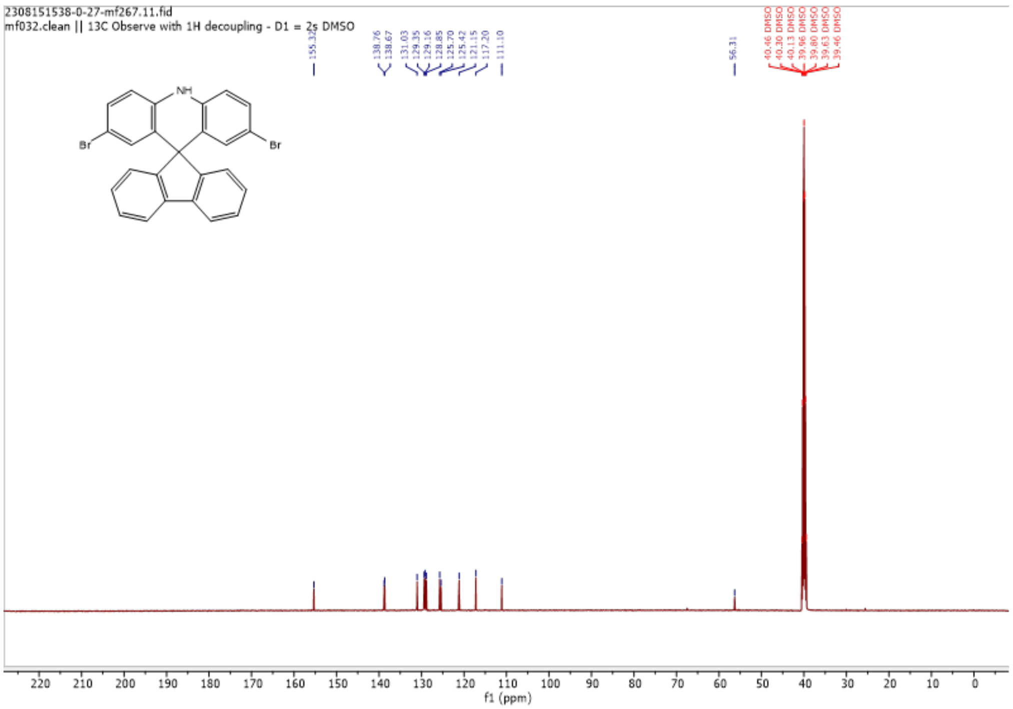

Figure **S15**. ^{13}C-NMR spectrum of **2,7-dibromo-10*H*-spiro[acridine-9,9'-fluorene] (SpAc-Br)** in DMSO-d_6.

a)

b)

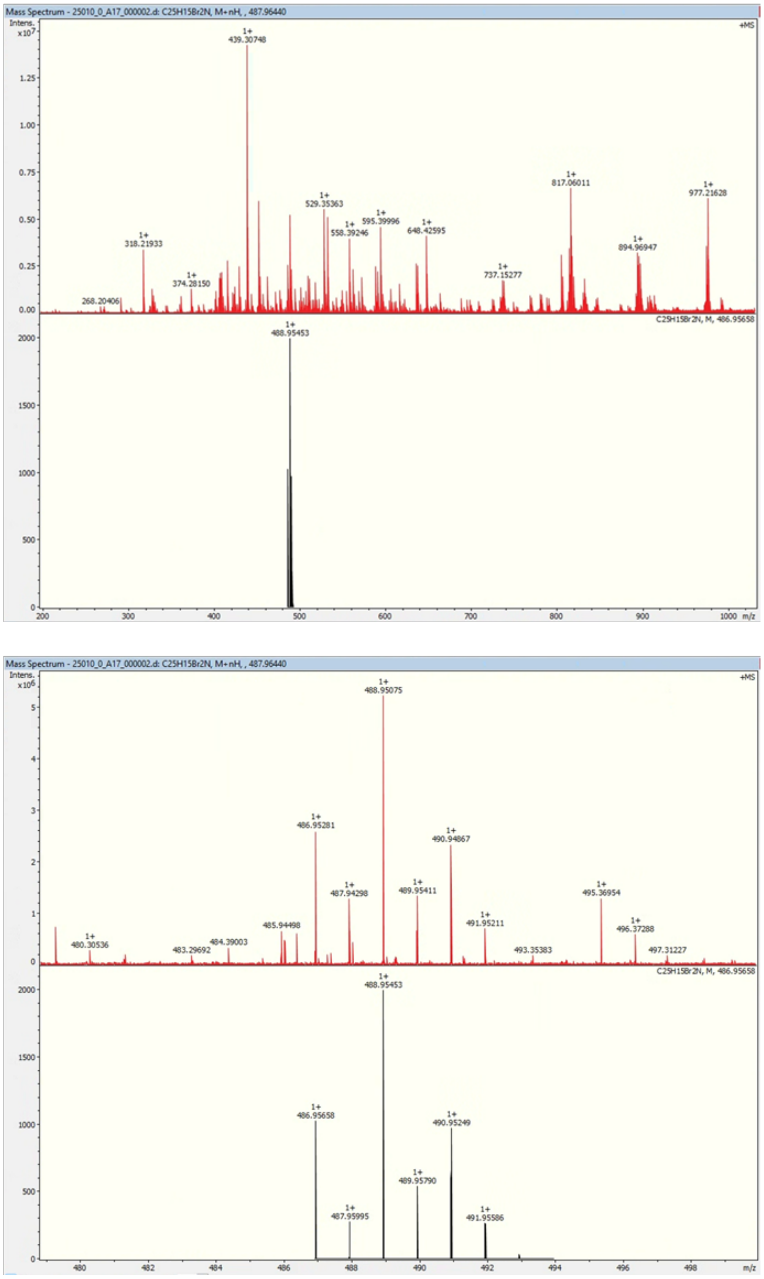

Figure **S16**. MALDI mass spectrum of **2,7-dibromo-10*H*-spiro[acridine-9,9'-fluorene] (SpAc-Br)**. a) Full spectrum, b) Zoom in on compound peak.

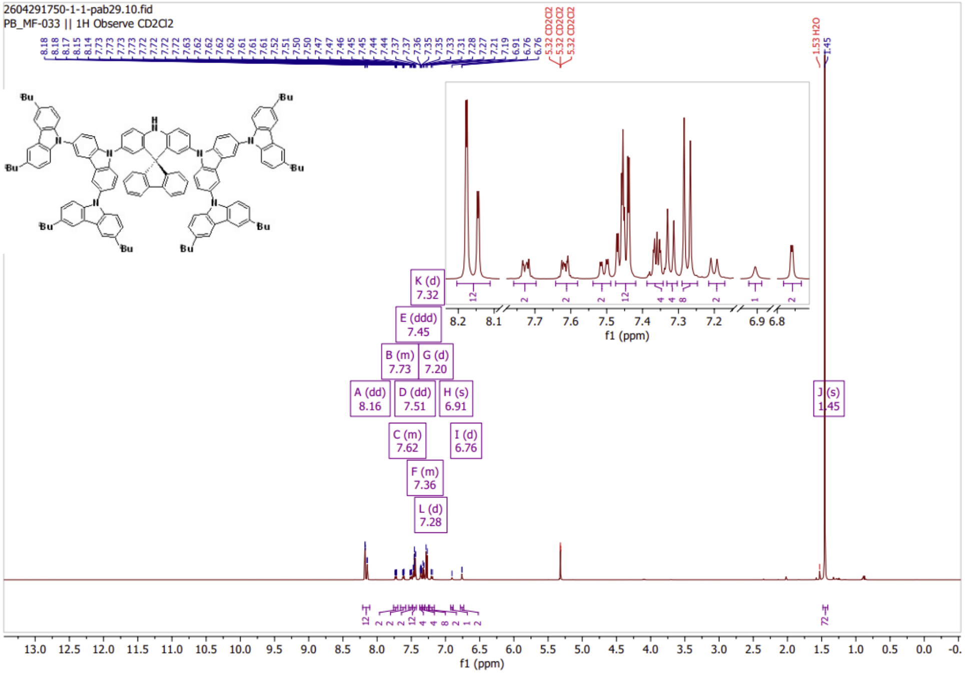

Figure **S17**. ^{1}H-NMR spectrum of **2,7-bis(3,3'',6,6''-tetra-*tert*-butyl-9'H-[9,3':6',9''-tercarbazol]-9'-yl)-10H-spiro[acridine-9,9'-fluorene] (SpAc-DCz)** in CD_2Cl_2.

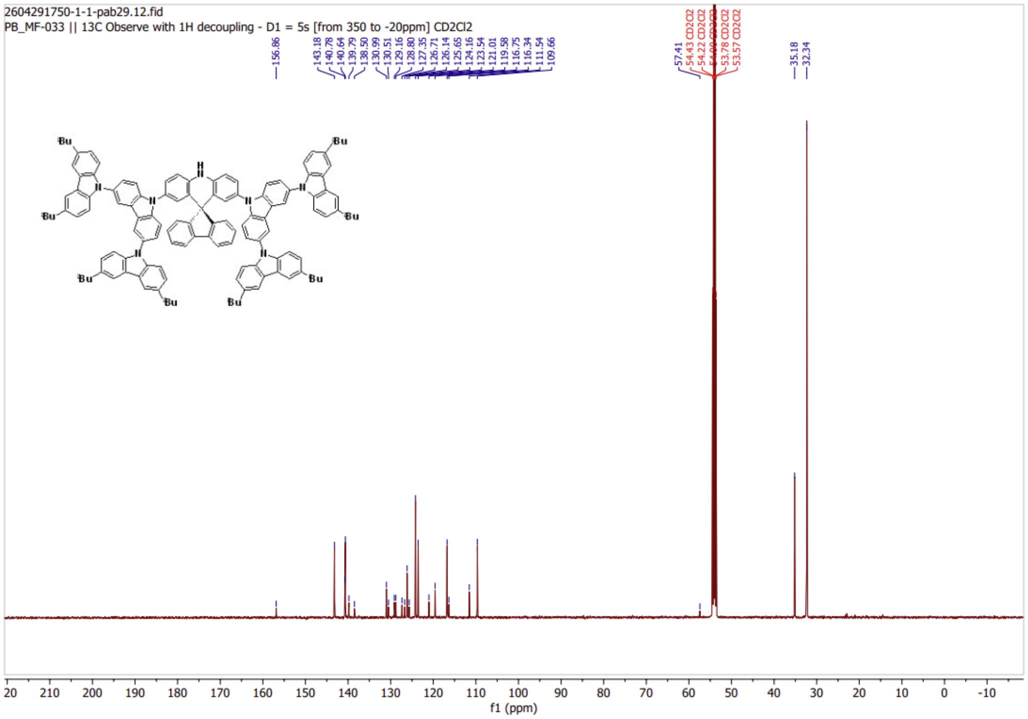

Figure **S18**. ^{13}C-NMR spectrum of **2,7-bis(3,3'',6,6''-tetra-*tert*-butyl-9'H-[9,3':6',9''-tercarbazol]-9'-yl)-10H-spiro[acridine-9,9'-fluorene] (SpAc-DCz)** in CD_2Cl_2.

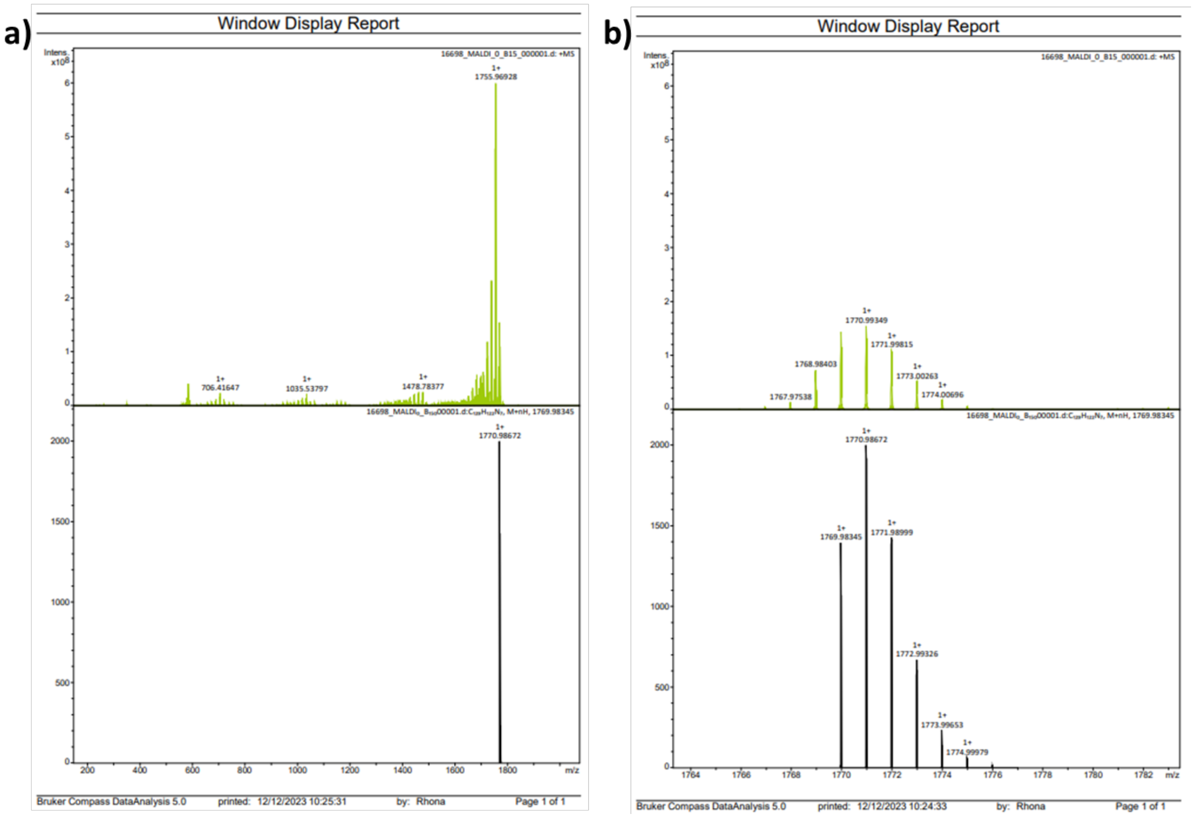

Figure **S19**. MALDI mass spectrum of **2,7-bis(3,3'',6,6''-tetra-*tert*-butyl-9'H-[9,3':6',9''-tercarbazol]-9'-yl)-10H-spiro[acridine-9,9'-fluorene] (SpAc-DCz)**. a) full spectrum, b) zoom in on compound peak.

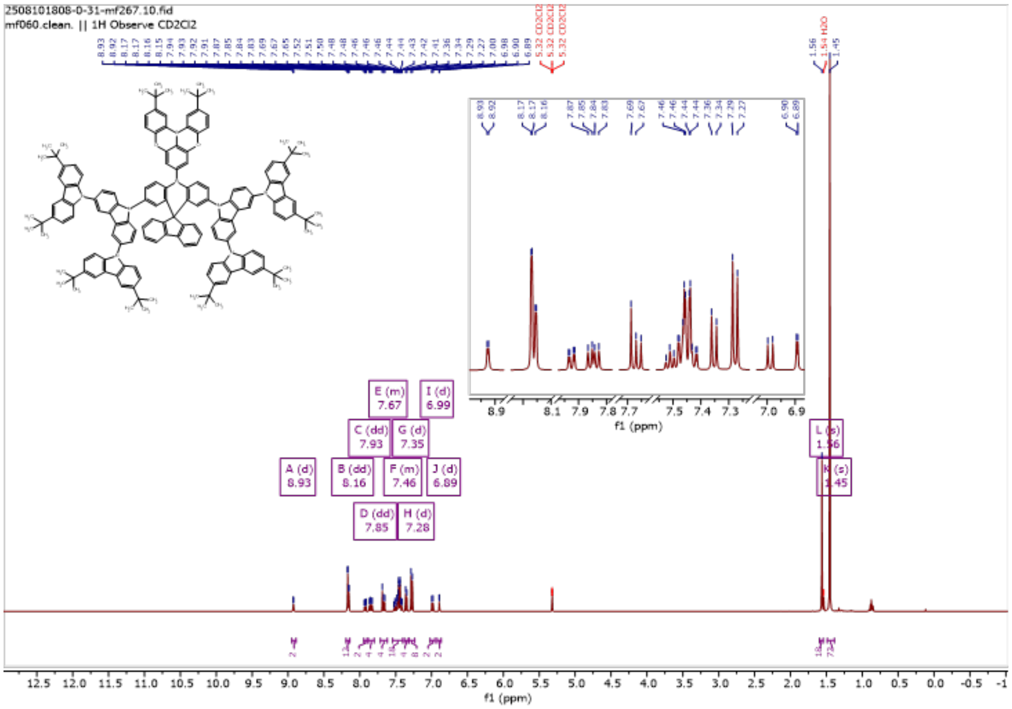

Figure **S20**. ^{1}H-NMR spectrum of **10-(2,12-di-*tert*-butyl-5,9-dioxa-13b-boranaphtho[3,2,1-de]anthracen-7-yl)-2,7-bis(3,3'',6,6''-tetra-*tert*-butyl-9'H-[9,3':6',9''-tercarbazol]-9'-yl)-10H-spiro[acridine-9,9'-fluorene] (DOBNA-SpAc-DCz)** in CD_2Cl_2.

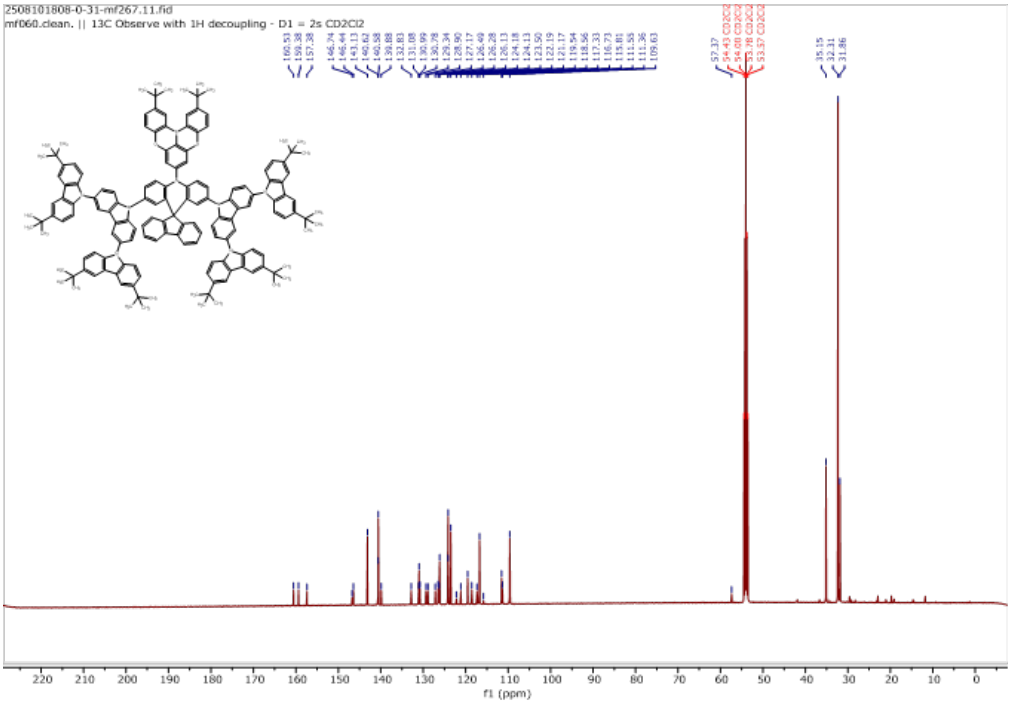

Figure **S21**. ^{13}C-NMR spectrum of **10-(2,12-di-*tert*-butyl-5,9-dioxa-13b-boranaphtho[3,2,1-de]anthracen-7-yl)-2,7-bis(3,3'',6,6''-tetra-*tert*-butyl-9'H-[9,3':6',9''-tercarbazol]-9'-yl)-10H-spiro[acridine-9,9'-fluorene] (DOBNA-SpAc-DCz)** in CD_2Cl_2.

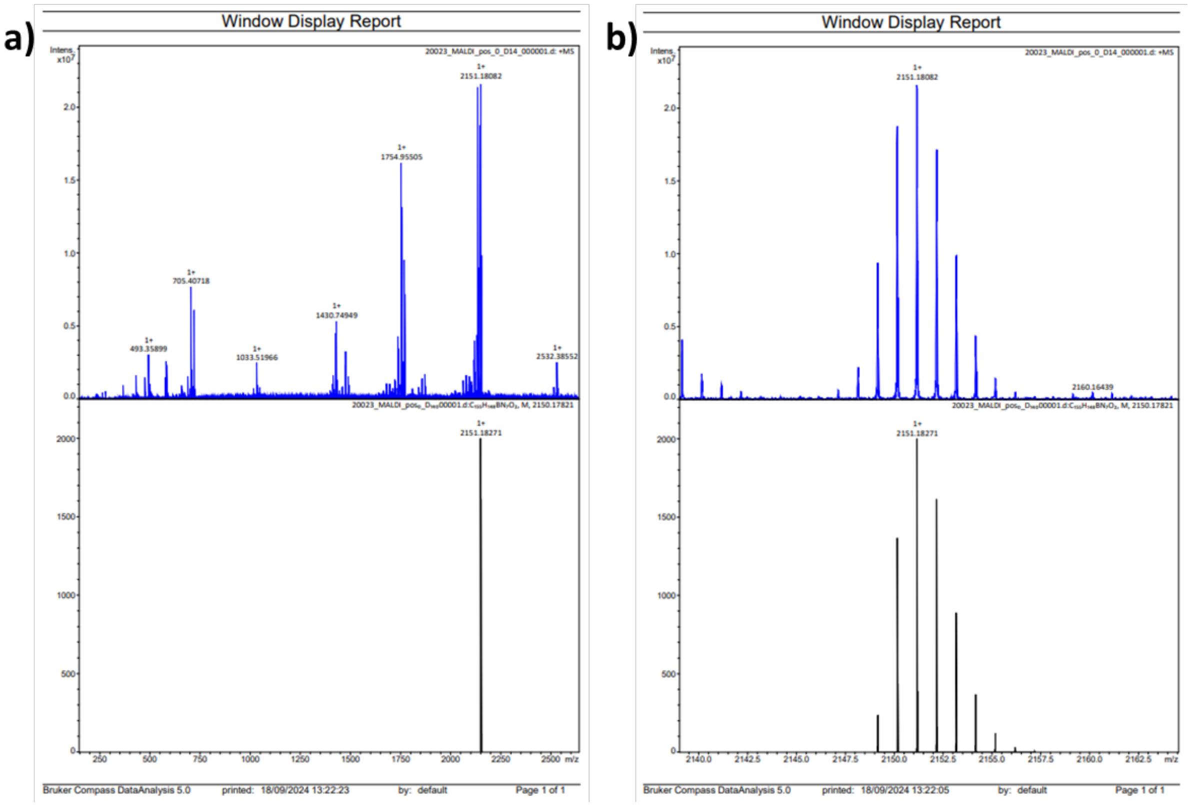

Figure **S22**. MALDI mass spectrum of **10-(2,12-di-*tert*-butyl-5,9-dioxa-13b-boranaphtho[3,2,1-de]anthracen-7-yl)-2,7-bis(3,3'',6,6''-tetra-*tert*-butyl-9'H-[9,3':6',9''-tercarbazol]-9'-yl)-10H-spiro[acridine-9,9'-fluorene] (DOBNA-SpAc-DCz).** a) full spectrum, b) zoom in on compound peak.

<Sample Information>

Sample Name : mf060
Sample ID :
Method Filename : 100% THF 20 mins 280nm -2ml-min new-please use.lcm
Batch Filename : DiKTaSe.lcb
Vial # : 1-29 Sample Type : Unknown
Injection Volume : 50 uL
Date Acquired : 14/01/2026 21:01:17 Acquired by : System Administrator
Date Processed : 14/01/2026 21:21:20 Processed by : System Administrator

<Chromatogram>

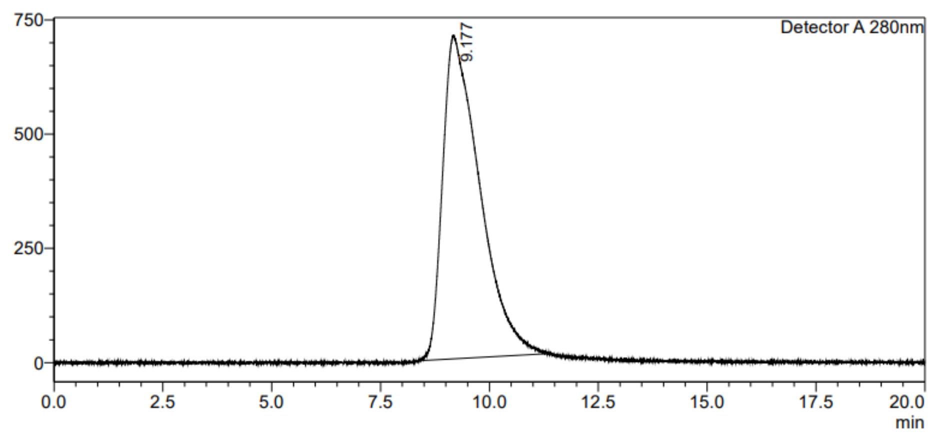

<Peak Table>

Detector A 280nm

Peak#	Ret. Time	Area	Height	Area%	Area/Height	Width at 5% Height
1	9.177	41871453	706122	100.000	59.298	2.083
Total		41871453	706122	100.000		

Figure **S23**. HPLC-GPC trace of **DOBNA-SpAc-DCz**.

Analysis – mf060			
**Element**	**Expected %**	**Found (1)**	**Found (2)**
**Carbon**	86.52	85.99	86.60
**Hydrogen**	6.93	6.04	6.19
**Nitrogen**	4.56	4.21	4.28

**Date completed**	04.02.2026
**Signature**	O. McCullough
**Comments**	

Figure **S24**. Elemental analysis of **DOBNA-SpAc-DCz**.

References

[1] H. Lim, H. J. Cheon, S. J. Woo, S. K. Kwon, Y. H. Kim, J. J. Kim, *Adv. Mater.* **2020**, 32, e2004083.
[2] K. Albrecht, K. Matsuoka, D. Yokoyama, Y. Sakai, A. Nakayama, K. Fujita, K. Yamamoto, *Chem. Commun.* **2017**, 53, 2439.
[3] M. J. Frisch, G. W. Trucks, H. B. Schlegel, G. E. Scuseria, M. A. Robb, J. R. Cheeseman, G. Scalmani, V. Barone, G. A. Petersson, H. Nakatsuji, X. Li, M. Caricato, A. V. Marenich, J. Bloino, B. G. Janesko, R. Gomperts, B. Mennucci, H. P. Hratchian, J. V. Ortiz, A. F. Izmaylov, J. L. Sonnenberg, Williams, F. Ding, F. Lipparini, F. Egidi, J. Goings, B. Peng, A. Petrone, T. Henderson, D. Ranasinghe, V. G. Zakrzewski, J. Gao, N. Rega, G. Zheng, W. Liang, M. Hada, M. Ehara, K. Toyota, R. Fukuda, J. Hasegawa, M. Ishida, T. Nakajima, Y. Honda, O. Kitao, H. Nakai, T. Vreven, K. Throssell, J. A. Montgomery Jr., J. E. Peralta, F. Ogliaro, M. J. Bearpark, J. J. Heyd, E. N. Brothers, K. N. Kudin, V. N. Staroverov, T. A. Keith, R. Kobayashi, J. Normand, K. Raghavachari, A. P. Rendell, J. C. Burant, S. S. Iyengar, J. Tomasi, M. Cossi, J. M. Millam, M. Klene, C. Adamo, R. Cammi, J. W. Ochterski, R. L. Martin, K. Morokuma, O. Farkas, J. B. Foresman, D. J. Fox, Wallingford, CT 2016.
[4] C. Adamo, V. Barone, *J. Chem. Phys.* **1999**, 110, 6158.
[5] G. A. Petersson, M. A. Al-Laham, *J. Chem. Phys.* **1991**, 94, 6081.
[6] S. Grimme, J. Antony, S. Ehrlich, H. Krieg, *J. Chem. Phys.* **2010**, 132, 154104.
[7] R. Dennington, T. A. Keith, J. M. Millam, Semichem Inc., Shawnee Mission, KS, 2016.
[8] S. Hirata, M. Head-Gordon, *Chem. Phys. Lett.* **1999**, 314, 291.
[9] X. Gao, S. Bai, D. Fazzi, T. Niehaus, M. Barbatti, W. Thiel, *J. Chem. Theory Comput.* **2017**, 13, 515.
[10] W. Humphrey, A. Dalke, K. Schulten, *Journal of Molecular Graphics* **1996**, 14, 33.
[11] O. S. Lee, E. Zysman-Colman, *Digichem (version 6) InSilico Computing*, St Andrews, Scotland, **2024**.
[12] O. Lee, M. Gather, E. Zysman-Colman, *Digital Discovery* **2024**, 3, 1695.
[13] N. M. O'Boyle, A. L. Tenderholt, K. M. Langner, *J. Comput. Chem.* **2008**, 29, 839.
[14] J. E. Stone, in *Faculty of the Graduate School of the University of Missouri - Rolla*, Vol. Master of Science in Computer Science, University of Missouri - Rolla, 1998.
[15] J. D. Hunter, *Computing in Science & Engineering* **2007**, 9, 90.
[16] N. M. O'Boyle, M. Banck, C. A. James, C. Morley, T. Vandermeersch, G. R. Hutchison, *Journal of Cheminformatics* **2011**, 3, 1.
[17] N. M. O'Boyle, G. R. Hutchison, *Chemistry Central Journal* **2008**, 2, 24.
[18] G. A. Crosby, J. N. Demas, *J. Phys. Chem.* **1971**, 75, 991.
[19] W. H. Melhuish, *J. Phys. Chem.* **1961**, 65, 229.
[20] V. V. Pavlishchuk, A. W. Addison, *Inorg. Chim. Acta* **2000**, 298, 97.
[21] Y. Tsuchiya, S. Diesing, F. Bencheikh, Y. Wada, P. L. Dos Santos, H. Kaji, E. Zysman-Colman, I. D. W. Samuel, C. Adachi, *J. Phys. Chem. A* **2021**, 125, 8074.
[22] H.-J. Tan, G.-X. Yang, Y.-L. Deng, C. Cao, J.-H. Tan, Z.-L. Zhu, W.-C. Chen, Y. Xiong, J.-X. Jian, C.-S. Lee, Q.-X. Tong, *Adv. Mater.* **2022**, 34, 2200537.

[23] J. Hwang, C. W. Koh, J. M. Ha, H. Y. Woo, S. Park, M. J. Cho, D. H. Choi, *ACS Appl. Mater. Interfaces* **2021**, 13, 61454.
